# Supplementary figures and images for: Small GTPase patterning: How to stabilise cluster coexistence
Source: PLoS One. 2019 Mar 7;14(3):e0213188. doi: 10.1371/journal.pone.0213188 (PMC6405054; doi:10.1371/journal.pone.0213188)

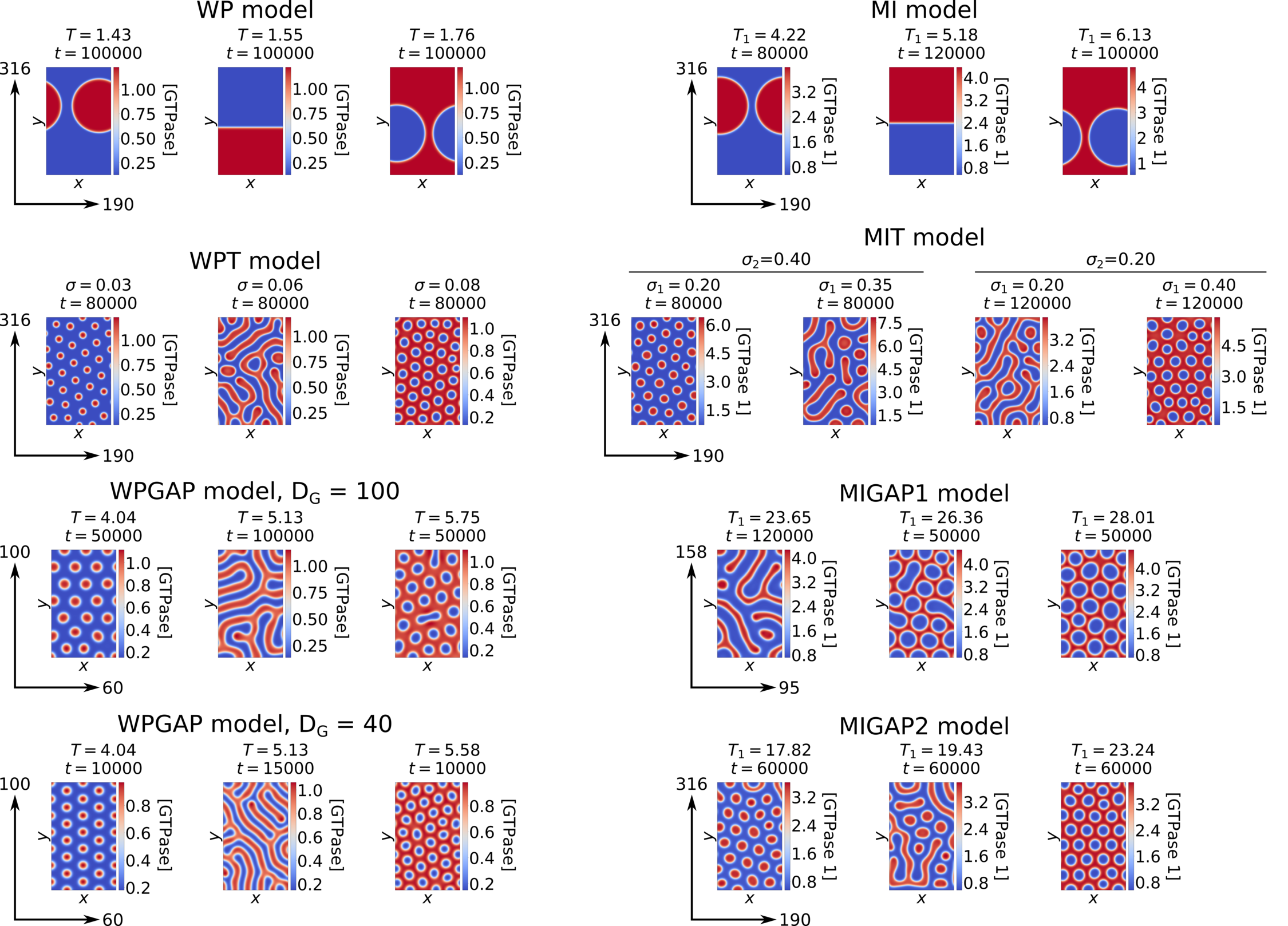

Supplement: S1 Fig — Simulations are as in Fig 2, but concentration ranges and time points at which simulations were stopped are indicated. (TIF) [file pone.0213188.s001.tif]

# WP model

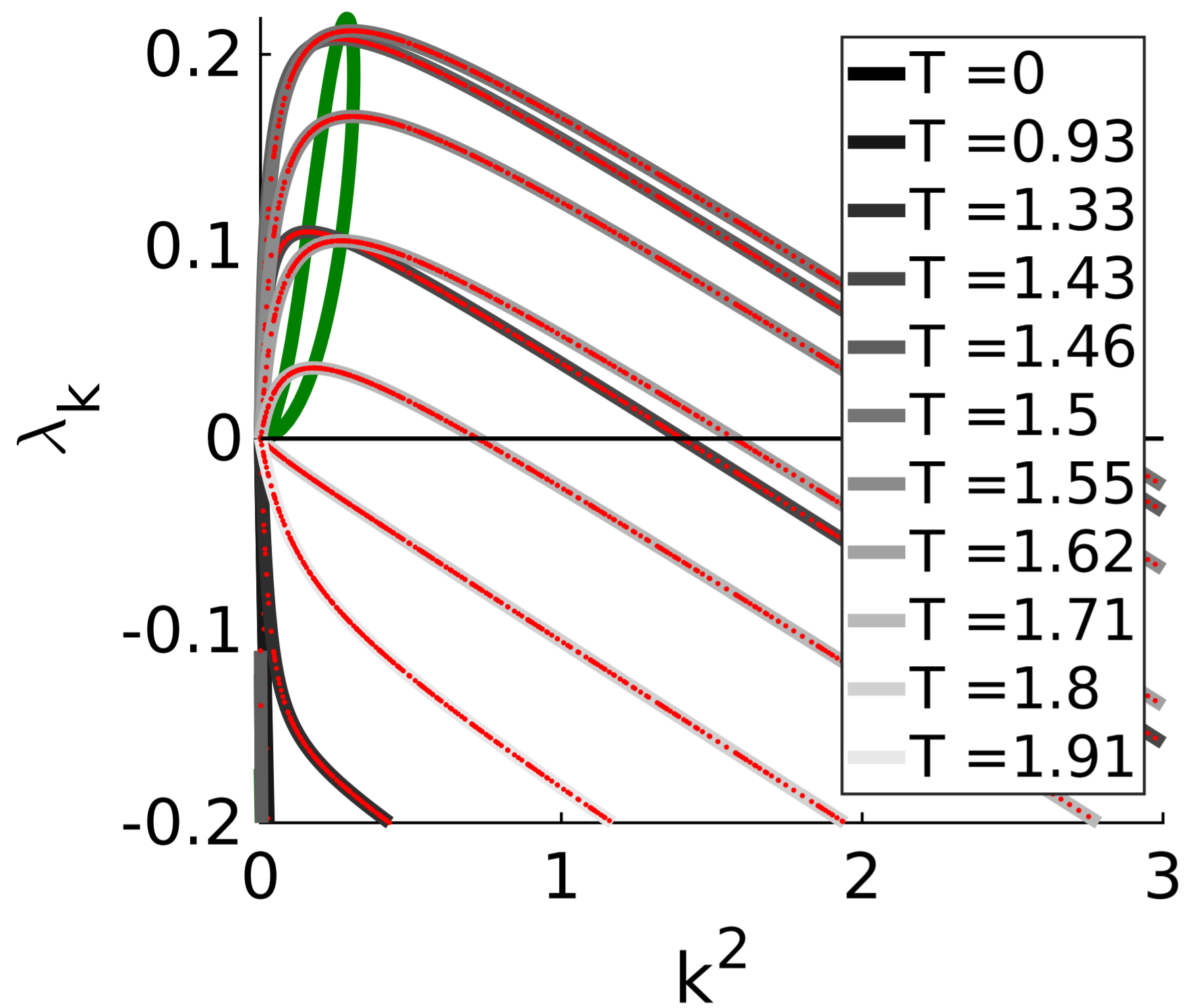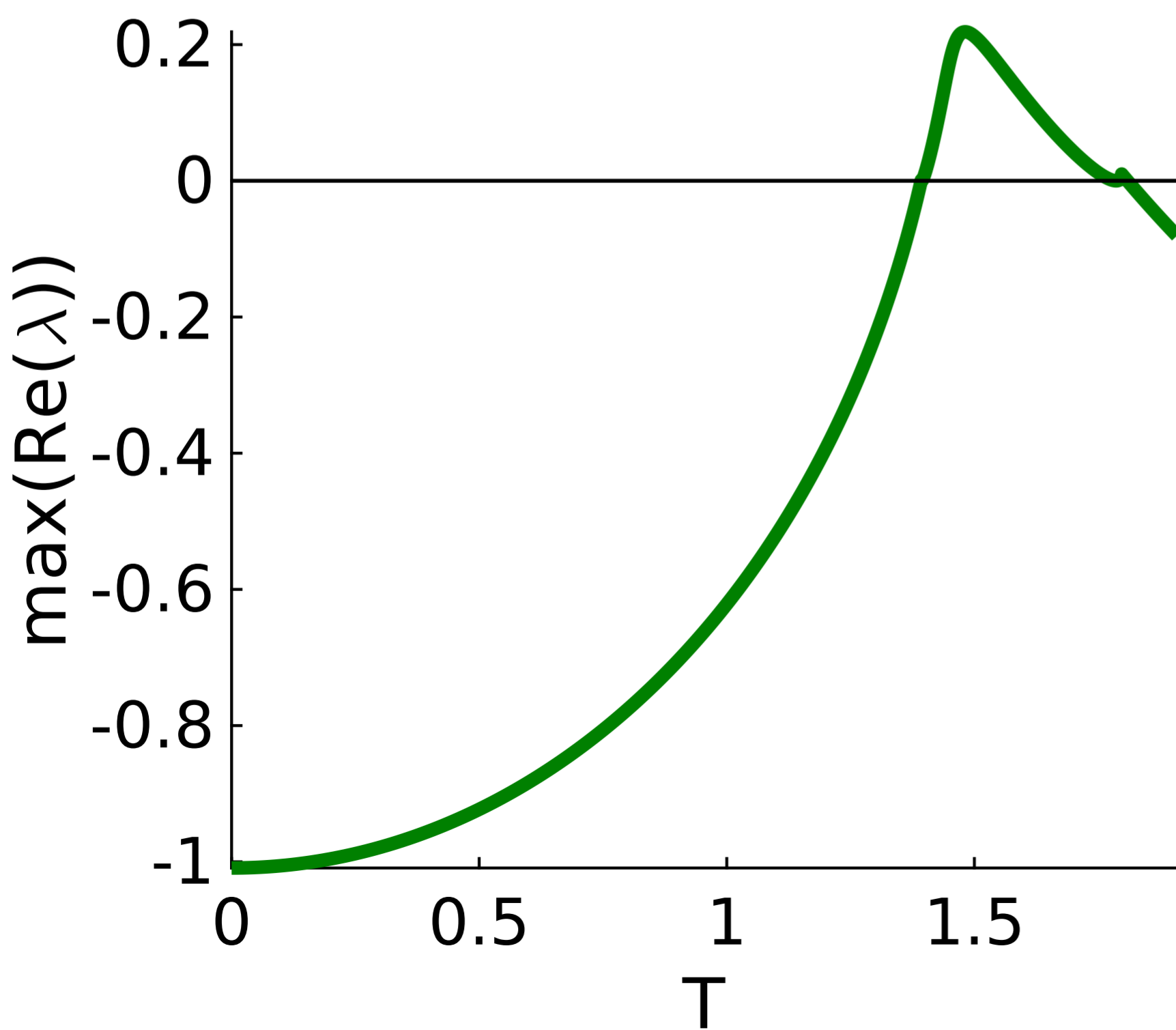

# WPT model

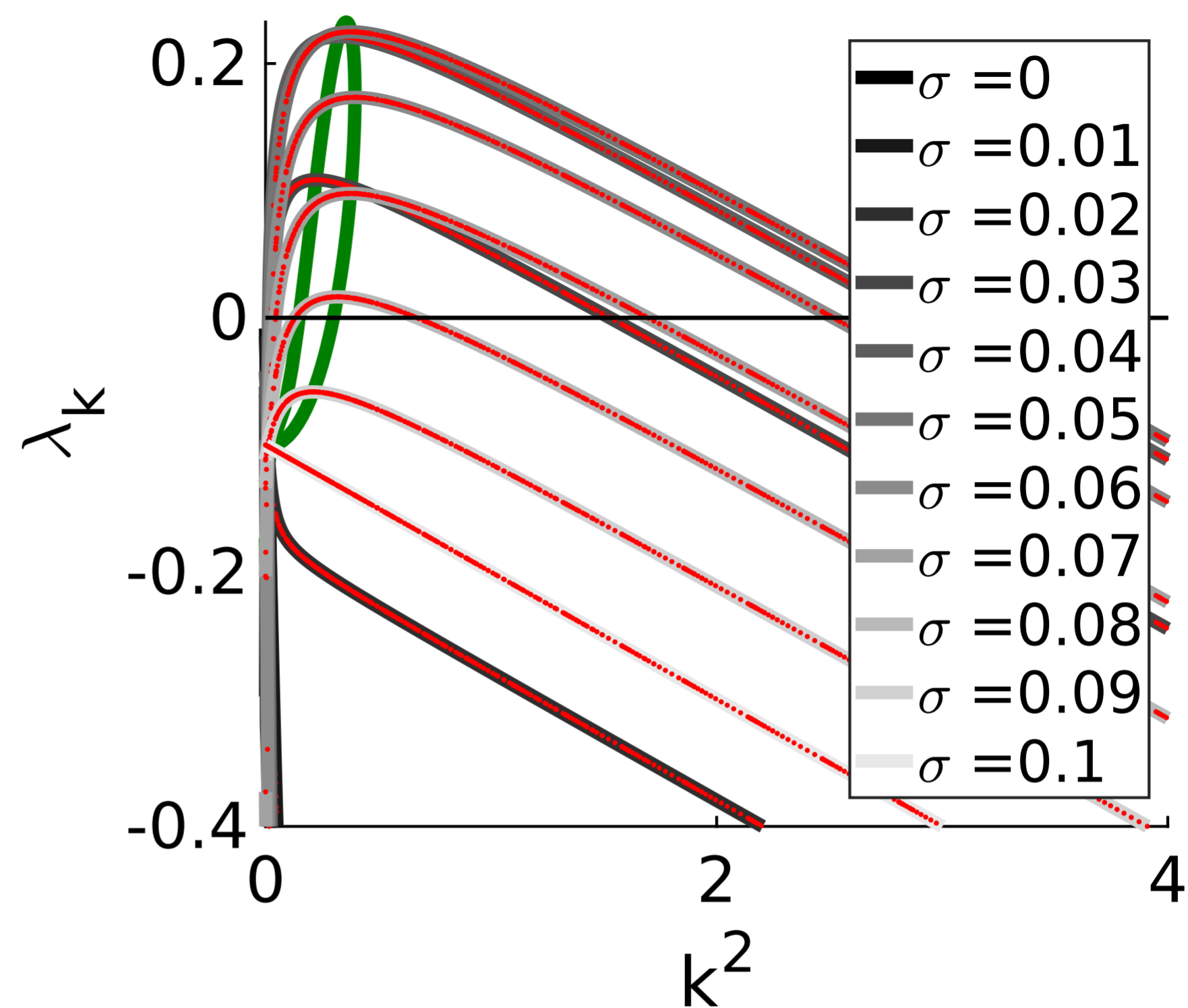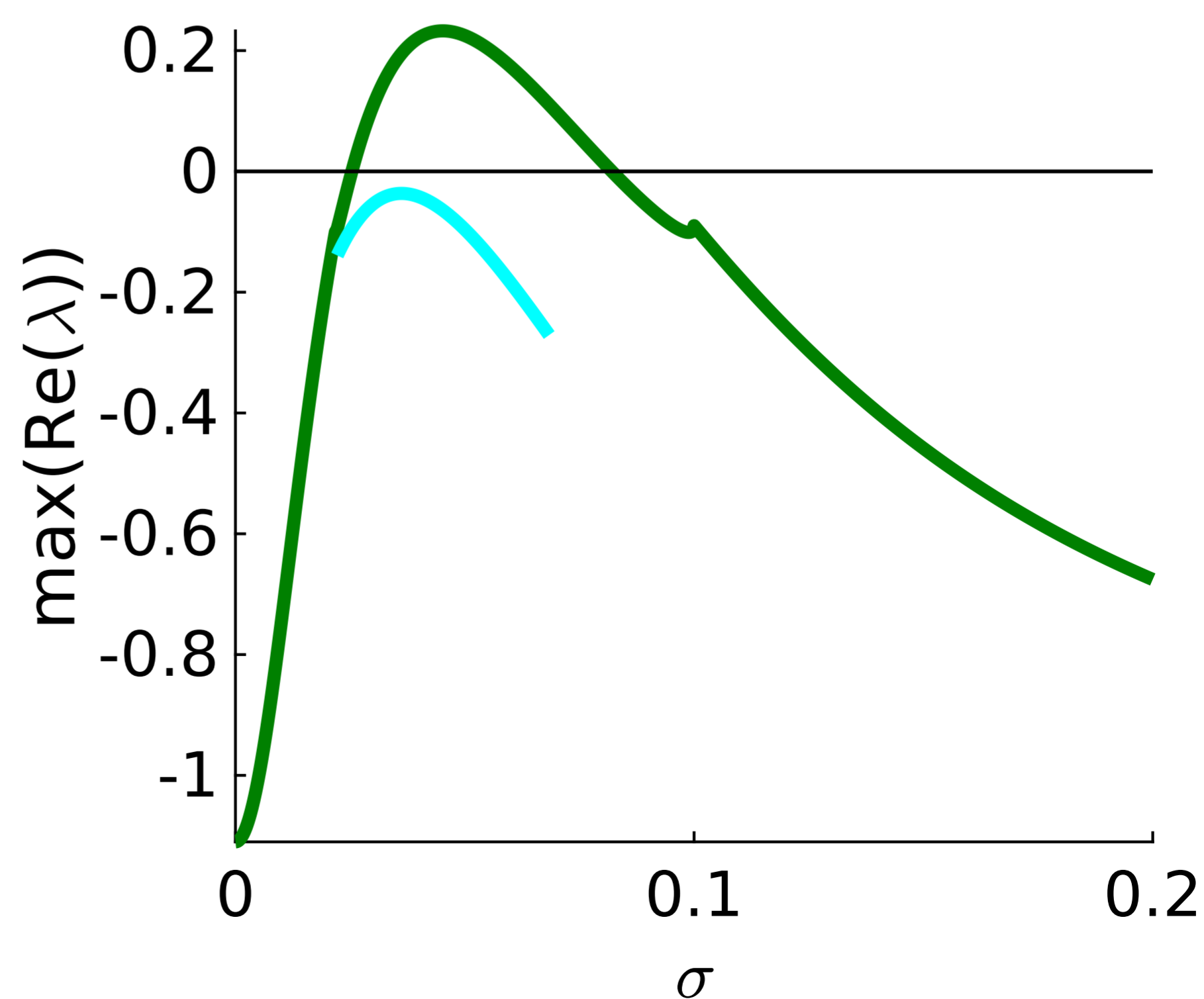

Supplement: S3 Fig — When the real part of at least one eigenvalue (λ) corresponding to a certain admissible wave number (k) is greater than zero the homogeneous state is unstable and a pattern forms. Top figures show λ as a function of k2 for various parameter values. Admissible wave numbers for the geometry of the simulations are indicated on these lines with red dots. Green lines show the maximum real part of λ as a function of total GTPase (T, WP model) or GTPase production rate (σ, WPT model), plotted both against these parameters (bottom) and against the squared wave numbers (top). Cyan lines indicate real parts of complex eigenvalues where present. (PDF) [file pone.0213188.s003.pdf]

Factor 10  
reduction

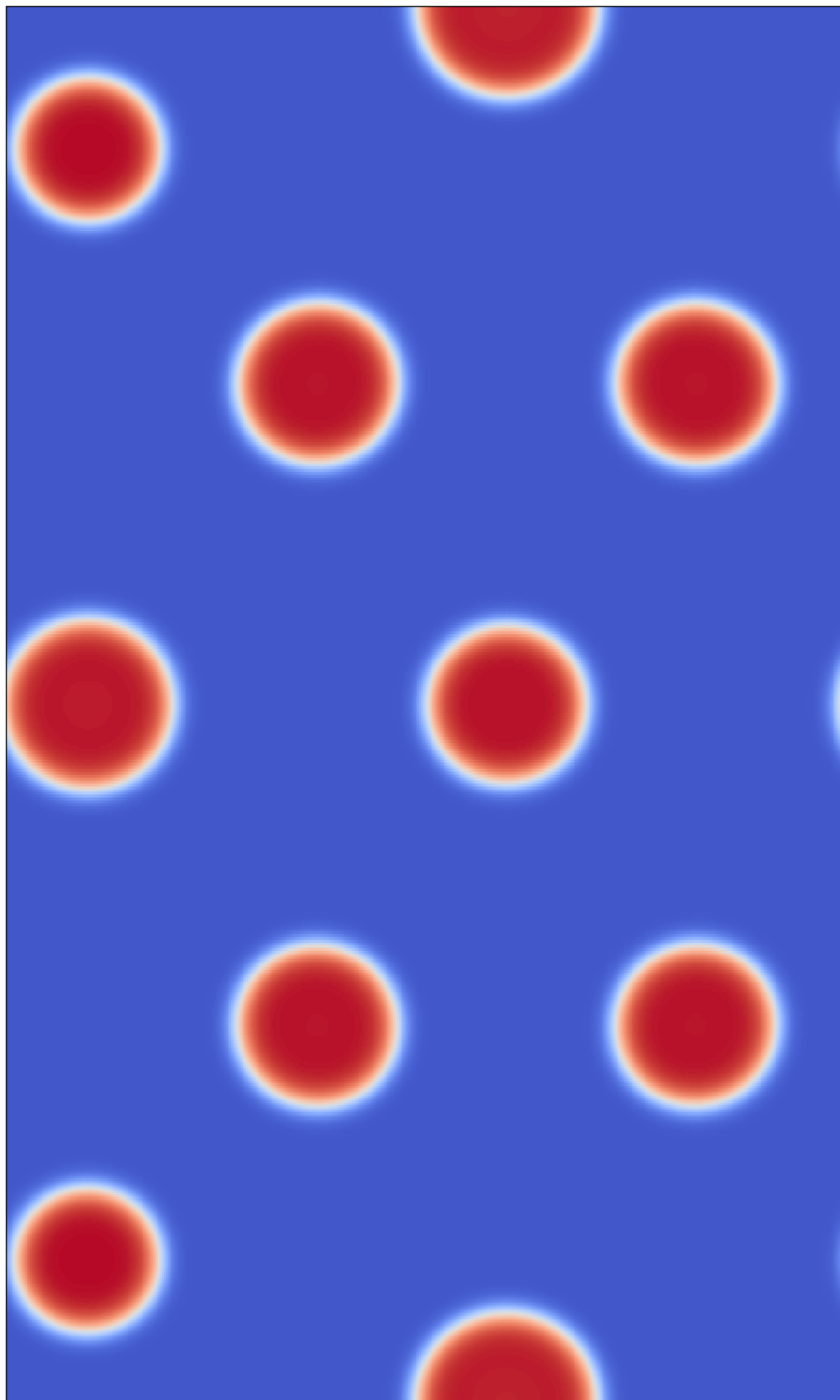

Factor 100  
reduction

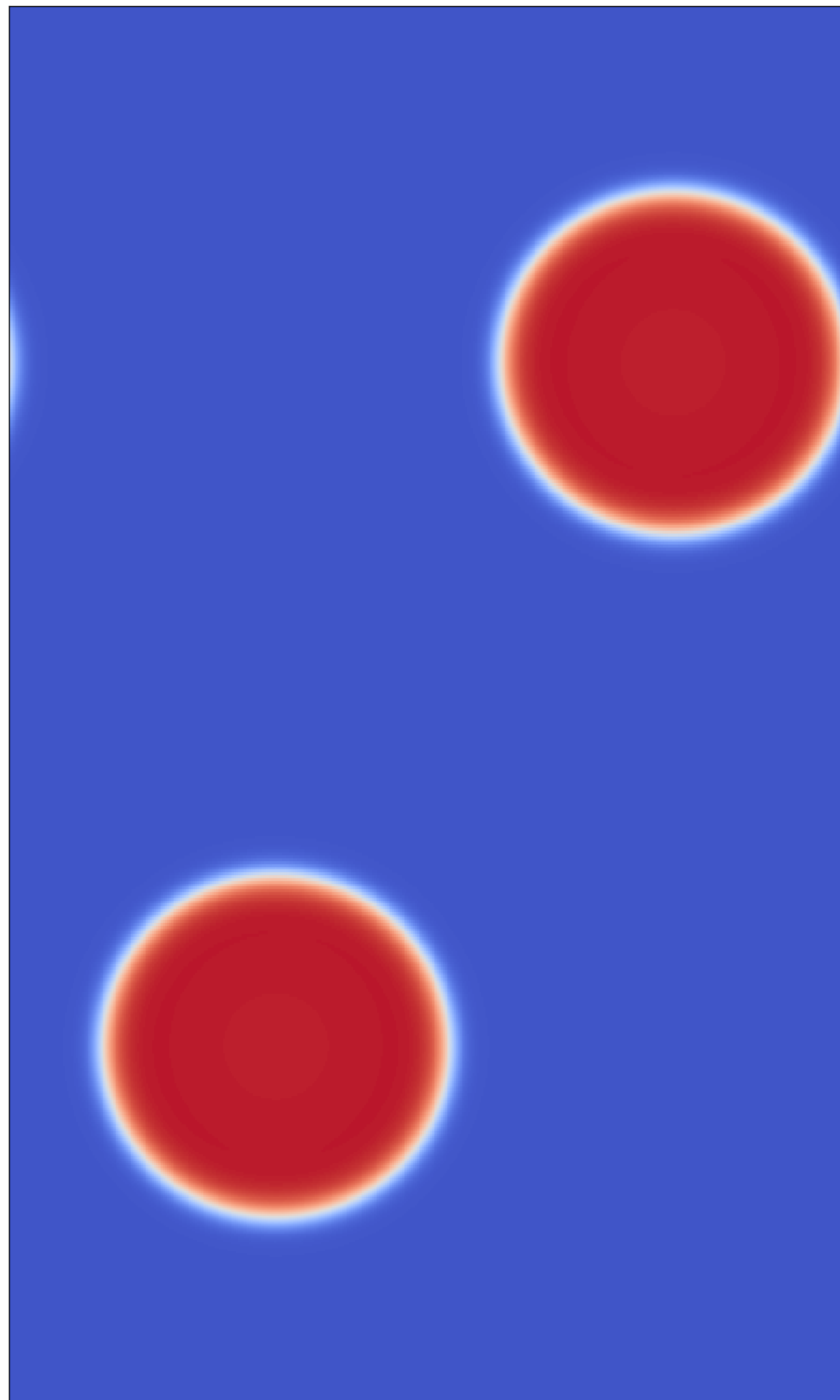

Factor 1000  
reduction

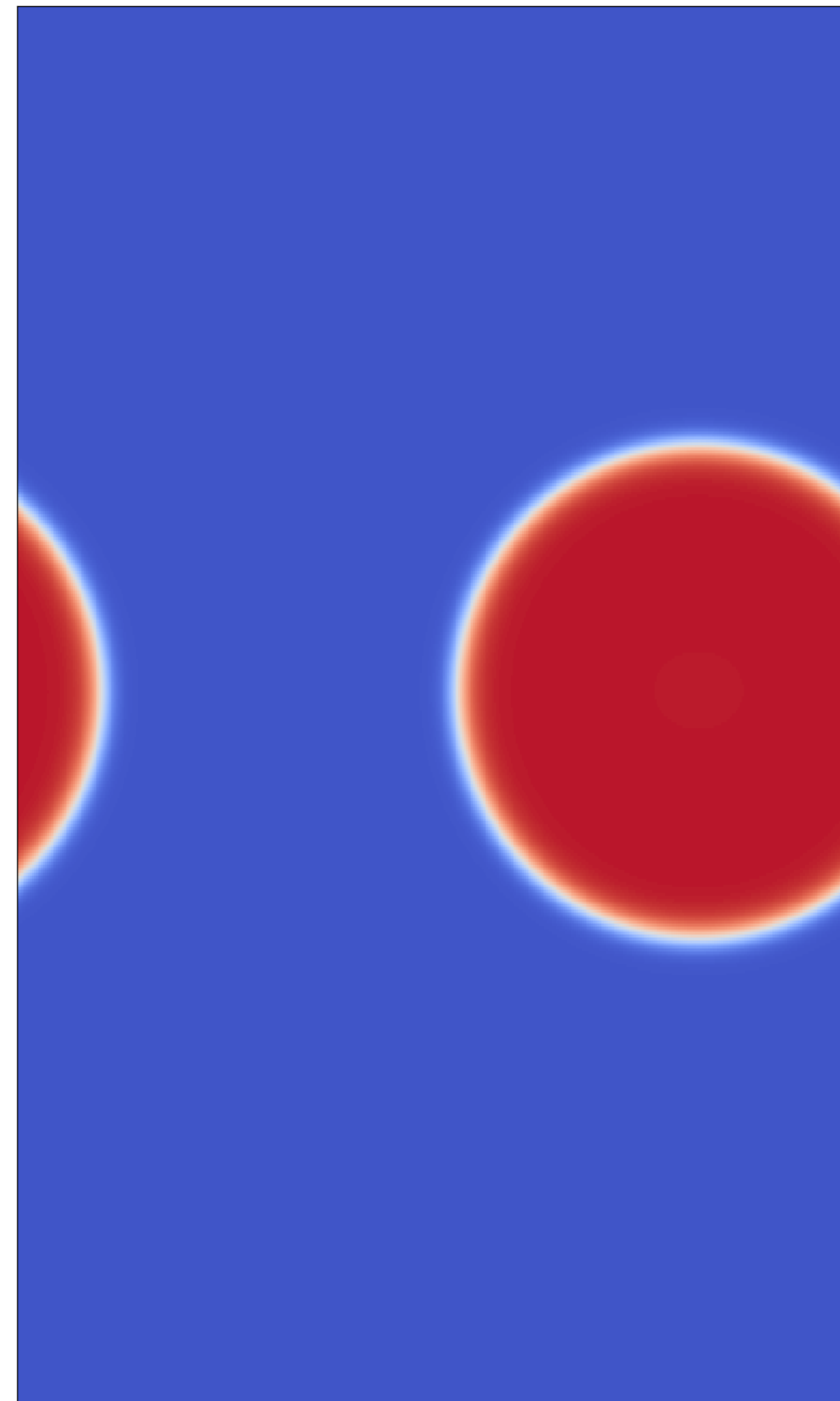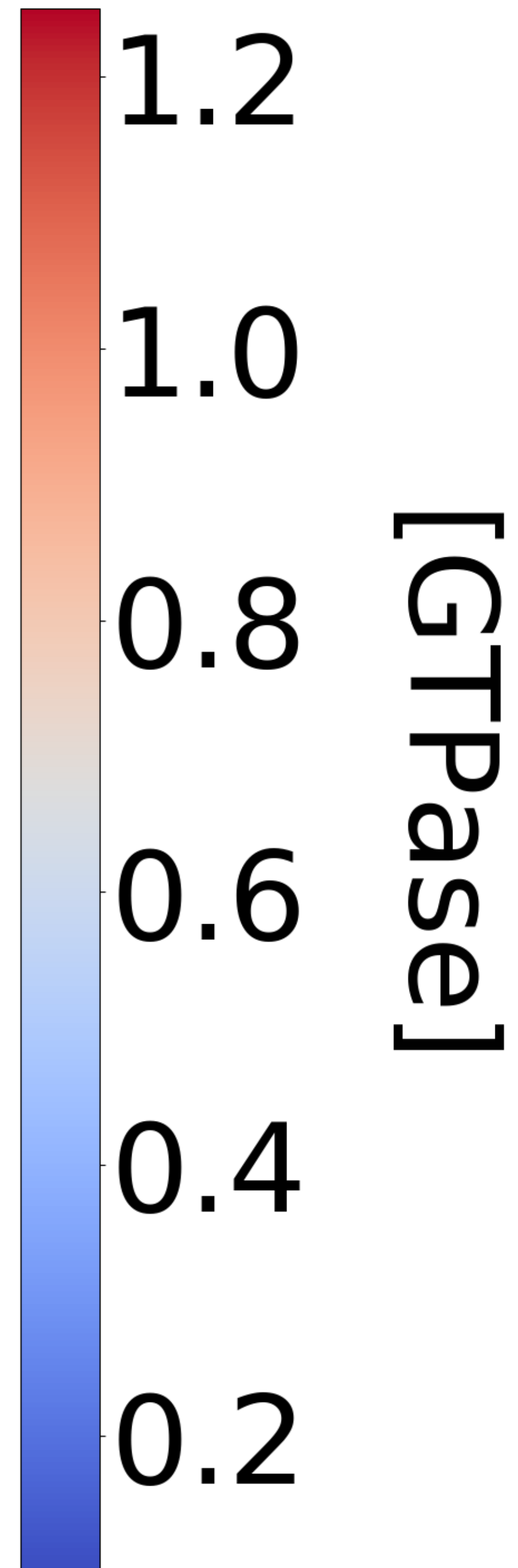

Supplement: S4 Fig — Steady state (t = 200000) active GTPase profiles generated by the WPT model with production (σ) and degradation (ξ) rates reduced by a factor 10, 100, and 1000 compared to default parameters. (PDF) [file pone.0213188.s004.pdf]

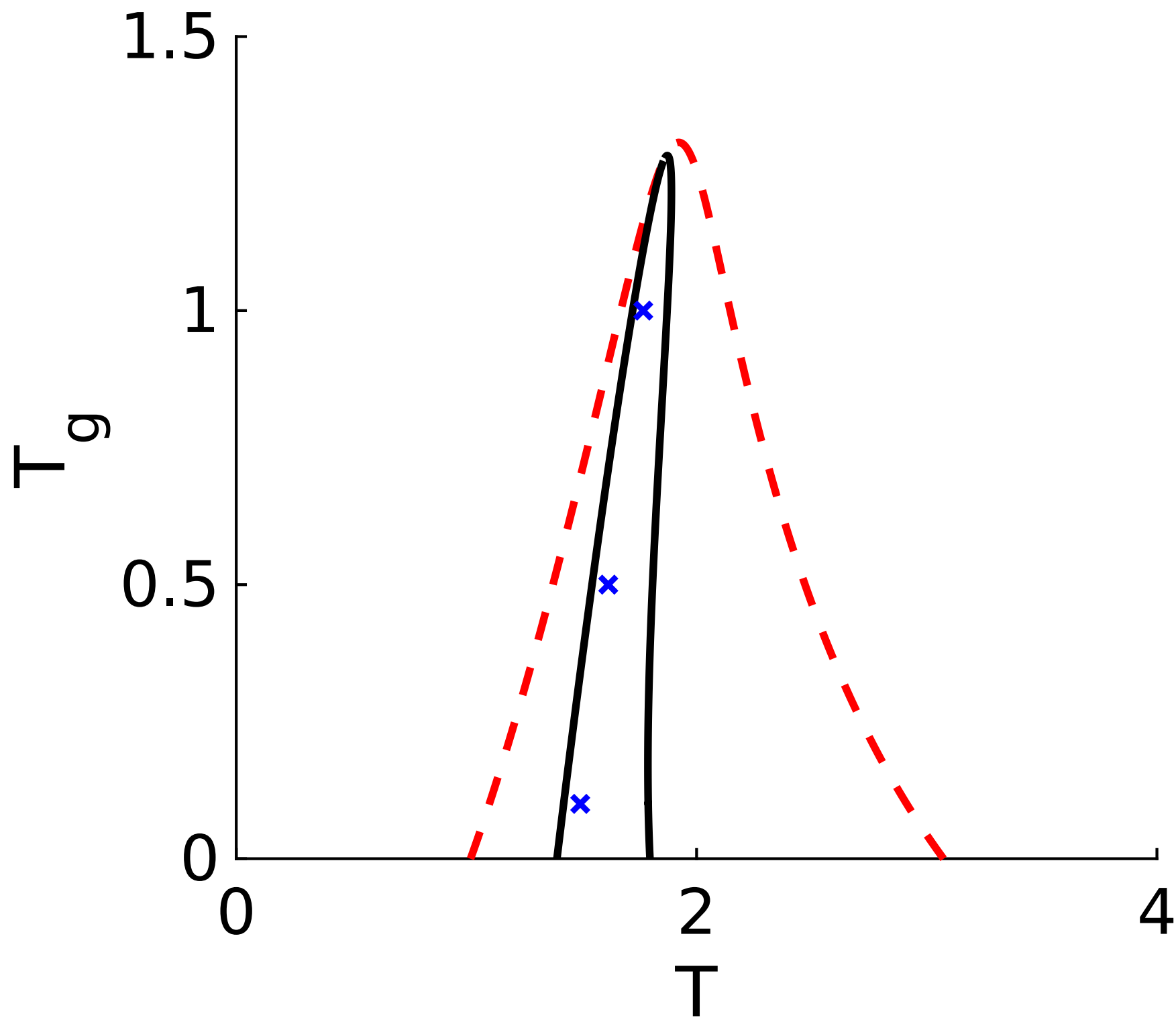

Supplement: S5 Fig — Crosses indicate parameter settings where trial simulations were performed. All simulations resulted in polarisation. (PDF) [file pone.0213188.s005.pdf]

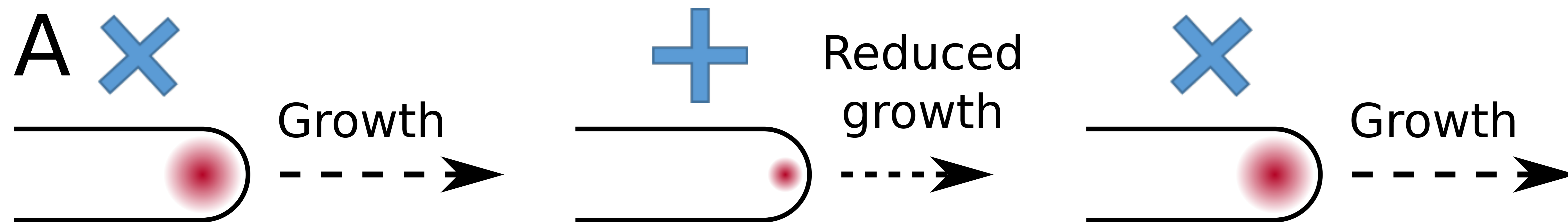

**B**

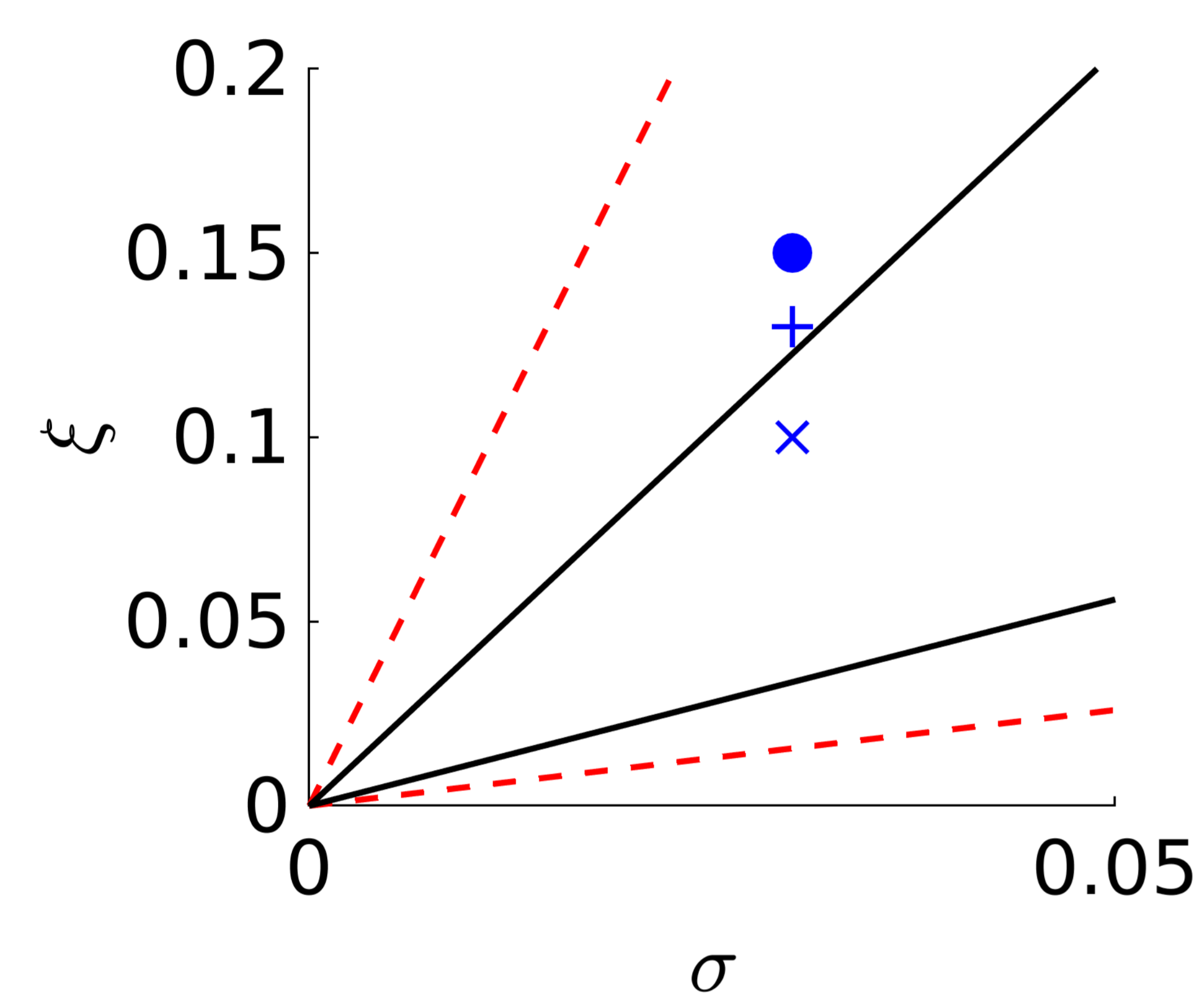

**C**

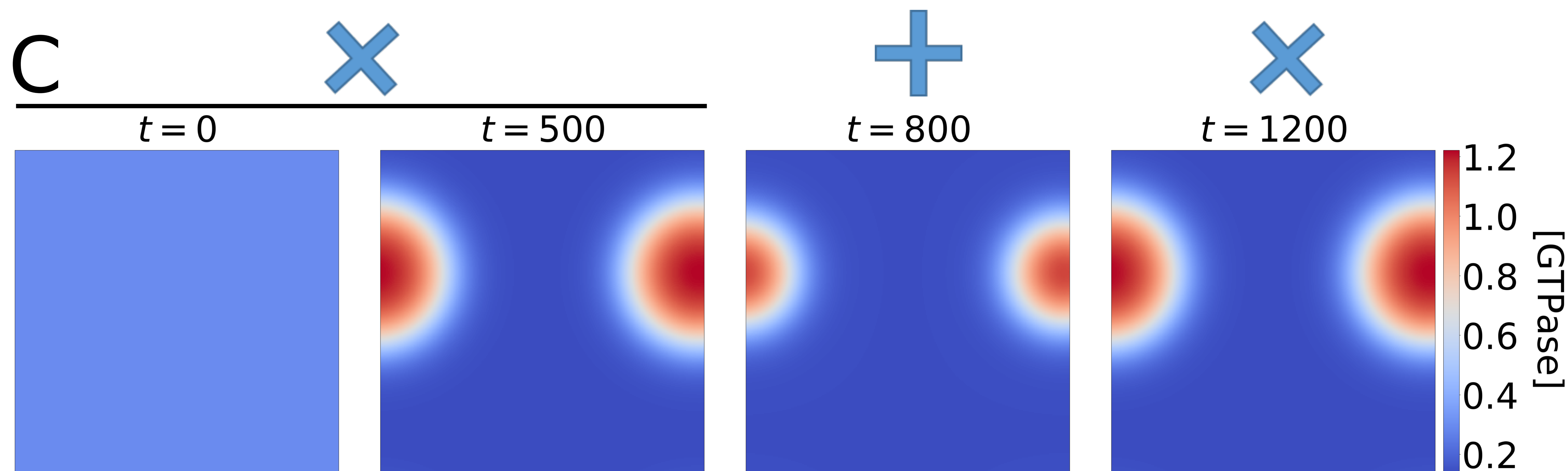

**D**

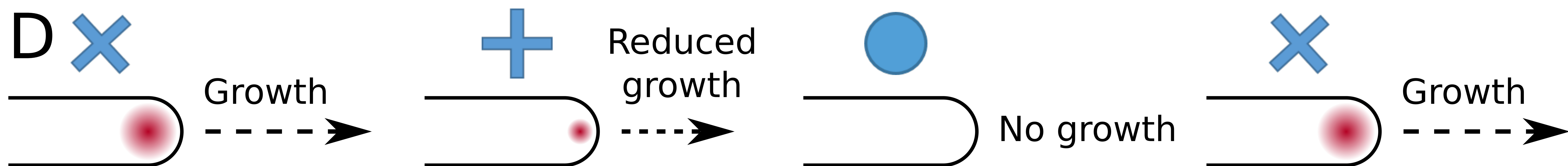

**E**

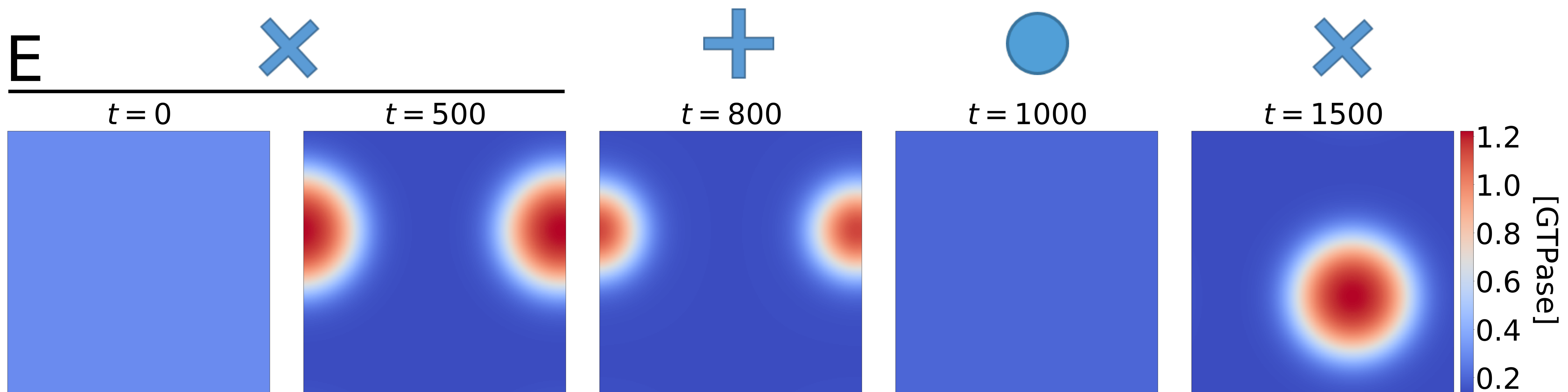

Supplement: S6 Fig — B: Bifurcation plot with symbols showing parameter settings used in the simulations, corresponding to symbols over simulations and cartoons. For meaning of lines see Fig 5. A: In pollen tubes, growth occurs in pulses as negative feedback during growth results in an oscillatory GTPase cluster at the growing tip. C: Simulation of a single active GTPase cluster with increase in ξ upon reaching steady state followed by a return to starting levels. D: In growing hyphae of A. gossypii, the tip growth complex sometimes disappears corresponding to a halt in growth, suggesting involvement of negative feedback. E: Simulation with two subsequent increases in ξ, followed by a return to the starting level. Symbols over simulations correspond to those in the bifurcation plot. Time points (t) of snapshots are indicated inside each plot. All simulations domains have periodic boundary conditions in both directions. Colourbars indicate active GTPase concentrations ([GTPase]). (PDF) [file pone.0213188.s006.pdf]
