# Supplementary material for: Small GTPase patterning: How to stabilise cluster coexistence
Source: PLoS One. 2019 Mar 7;14(3):e0213188. doi: 10.1371/journal.pone.0213188 (PMC6405054; doi:10.1371/journal.pone.0213188)
Supplement: S1 Table — (PDF) [file pone.0213188.s007.pdf]

Model	dx = dy	dt
WP	0.2	0.5
MI	0.25	0.2
WPT	0.2	0.5
MIT	0.2	0.2
WPGAP, $D_G = 100$	0.25	0.2
WPGAP, $D_G = 40$	0.2	0.1
MIGAP1	0.25	0.2
MIGAP2	0.25	0.1
Tip growth simulations	0.05	0.05
