# Supplementary material for: Small GTPase patterning: How to stabilise cluster coexistence
Source: PLoS One. 2019 Mar 7;14(3):e0213188. doi: 10.1371/journal.pone.0213188 (PMC6405054; doi:10.1371/journal.pone.0213188)
Supplement: S2 Fig — Active GTPase concentration profiles at various time points for WP model simulations from Fig 2. Figures show that in all three cases, pattern formation starts with gradual coarsening throughout the simulation domain (reminiscent of spinodal decomposition). All sharply delineated gaps at t = 1700 arise as continuous amplifications of an earlier pattern, even though, inside a limited time window, gaps seem to appear one after another (superficially resembling nucleation and growth). (PDF) [file pone.0213188.s002.pdf]

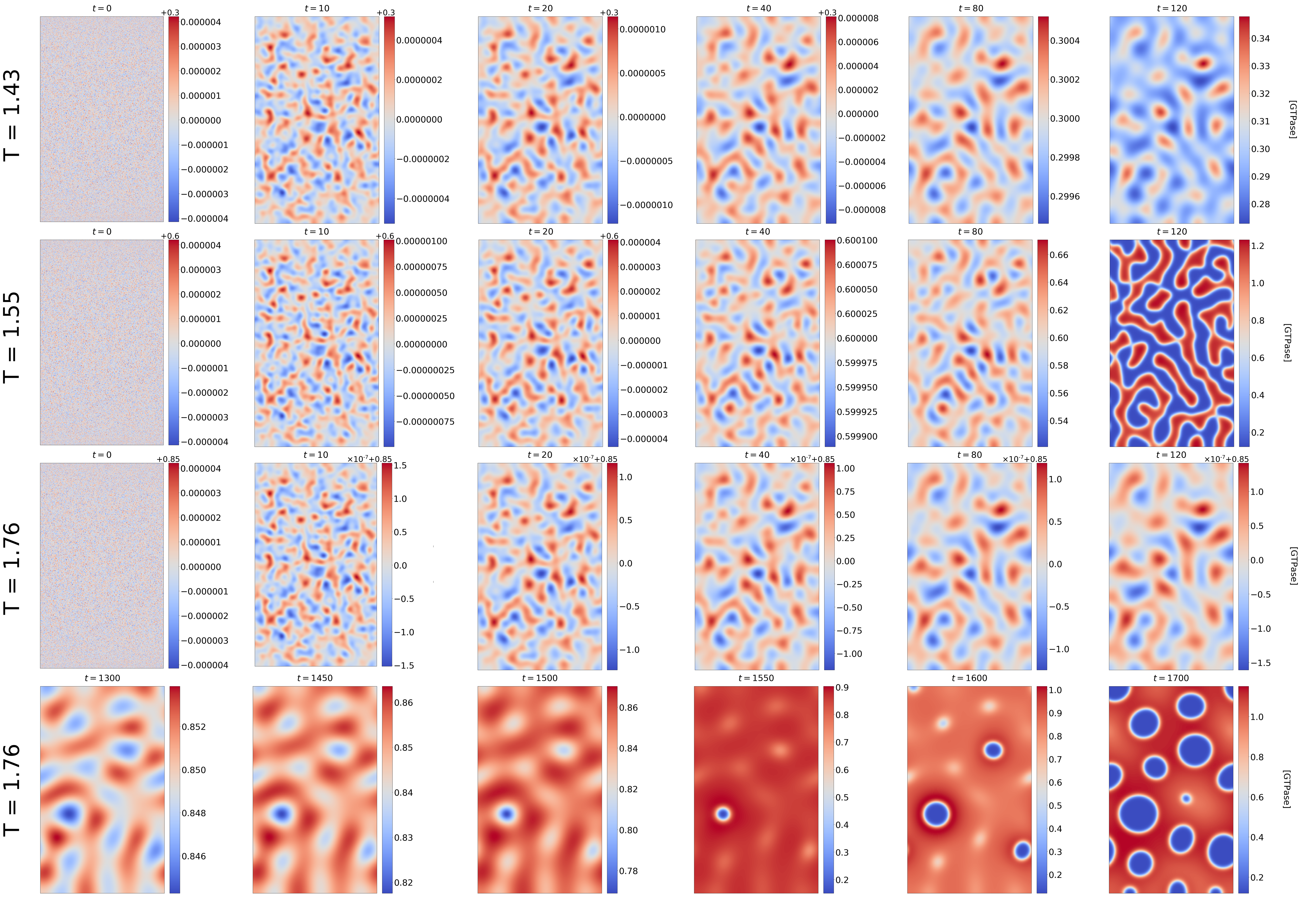

T = 1.43
T = 1.55
T = 1.76
T = 1.76
$t = 0$
$t = 10$
$t = 20$
$t = 40$
$t = 80$
$t = 120$
$t = 1300$
$t = 1450$
$t = 1500$
$t = 1550$
$t = 1600$
$t = 1700$
[GTPase]
