# Supplementary material for: Small GTPase patterning: How to stabilise cluster coexistence
Source: PLoS One. 2019 Mar 7;14(3):e0213188. doi: 10.1371/journal.pone.0213188 (PMC6405054; doi:10.1371/journal.pone.0213188)
Supplement: S1 Appendix — Including non-dimensionalisation, derivation of homogeneous steady states, details of LSA and LPA methods, analysis of the WPT model with degradation of active GTPase, detailed derivation of the ODE models, and the quasi steady state approximation used for the ODE model with GAP feedback. (PDF) [file pone.0213188.s008.pdf]

Supplementary material for: Small GTPase patterning: How to stabilise cluster coexistence

Bas Jacobs, Jaap Molenaar, Eva E. Deinum

Contents

1 Non-dimensionalisation **3**
1.1 Non-dimensionalisation of the WP model 3
1.2 Non-dimensionalisation of the MI model 4
1.3 Non-dimensionalisation of the WPT model 4
1.4 Non-dimensionalisation of the MIT model 5
1.5 Non-dimensionalisation of the WPGAP model 6
1.6 Non-dimensionalisation of the MIGAP1 model 7
1.7 Non-dimensionalisation of the MIGAP2 model 8

2 Homogeneous steady states **9**

3 Linear stability analysis for two-component reaction-diffusion systems in two dimensions **11**
3.1 Linearisation and growth modes 11
3.2 Wave numbers 12
3.2.1 Periodic boundary conditions 13
3.2.2 Zero flux boundary conditions 14
3.2.3 Admissible wave numbers 14
3.3 One parameter bifurcation analysis 14
3.4 Hopf bifurcations 15
3.5 Two parameter bifurcation analysis 16

4 Local perturbation analysis **16**
4.1 LPA of the WP model 16
4.2 LPA of the MI model 17
4.3 LPA of the WPT model 18
4.4 LPA of the MIT model 19
4.5 LPA of the WPGAP model 20
4.6 LPA of the MIGAP1 model 21
4.7 LPA of the MIGAP2 model 22

5 WPT model with degradation of inactive GTPase **23**

6 **Derivation of ODE models** **25**
6.1 Basic ODE model 25
6.2 Compartmentalised ODE model 26
6.3 ODE model with GAP feedback 27

7 **WPT model with degradation of both active and inactive GTPase** **28**

8 **Quasi steady state approximation for GAPs in ODE model** **28**

References **31**

1 Non-dimensionalisation

1.1 Non-dimensionalisation of the WP model

The full dimension-carrying WP model is given by:

$$\begin{aligned}\frac{\partial u}{\partial t} &= bv + \gamma\frac{u^n}{K^n+u^n}v - \delta u + D_u\nabla^2 u \\ \frac{\partial v}{\partial t} &= -bv - \gamma\frac{u^n}{K^n+u^n}v + \delta u + D_v\nabla^2 v,\end{aligned} \tag{1}$$

where u and v are the concentrations of active and inactive GTPase, respectively, t is the time, b is a constant activation rate, δ is a constant inactivation rate, γ determines the strength of the feedback activation, K is the concentration of active GTPase at which the feedback activation is half its maximum, exponent n is a hill coefficient, and D_u and D_v are the diffusion coefficients of active and inactive GTPase, respectively. Due to mass conservation, the average total GTPase concentration T is constant:

$$T = \frac{1}{A}\iint_A (u+v)dxdy = \frac{\text{Total GTPase}}{A}, \tag{2}$$

where A is the domain area and x and y are the spatial coordinates. Non-dimensionalisation was performed as previously described (Holmes and Edelstein-Keshet, 2016). GTPase concentrations were scaled with K, time with δ, and space with D_u and δ. The resulting dimensionless variables are:

$$\tilde{u} = \frac{u}{K}, \quad \tilde{v} = \frac{v}{K}, \quad \tilde{t} = \delta t, \quad \tilde{x} = \frac{x\sqrt{\delta}}{\sqrt{D_u}}, \quad \tilde{y} = \frac{y\sqrt{\delta}}{\sqrt{D_u}}, \tag{3}$$

and the dimensionless parameters:

$$\tilde{T} = \frac{T}{K}, \quad \tilde{b} = \frac{b}{\delta}, \quad \tilde{\gamma} = \frac{\gamma}{\delta}, \quad \tilde{D}_v = \frac{D_v}{D_u}, \quad \tilde{A} = A\frac{\delta}{D_u}. \tag{4}$$

This makes the dimensionless total amount of GTPase:

$$\text{Total }\tilde{\text{G}}\text{TPase} = \frac{\text{Total GTPase}\cdot\delta}{D_u K}. \tag{5}$$

Dropping the tildes, we obtain the dimensionless WP model:

$$\begin{aligned}\frac{\partial u}{\partial t} &= bv + \gamma\frac{u^n}{1+u^n}v - u + \nabla^2 u \\ \frac{\partial v}{\partial t} &= -bv - \gamma\frac{u^n}{1+u^n}v + u + D_v\nabla^2 v,\end{aligned} \tag{6}$$

with dimensionless average total GTPase concentration:

$$T = \frac{1}{A}\iint_A (u+v)dxdy = \frac{\text{Total GTPase}}{A}. \tag{7}$$

1.2 Non-dimensionalisation of the MI model

The full dimension-carrying MI model is given by:

$$\begin{aligned}
\frac{\partial u_1}{\partial t} &= b_1 v_1 + \gamma_1 \frac{K_2^n}{K_2^n + u_2^n} v_1 - \delta u_1 + D_u \nabla^2 u_1 \\
\frac{\partial v_1}{\partial t} &= -b_1 v_1 - \gamma_1 \frac{K_2^n}{K_2^n + u_2^n} v_1 + \delta u_1 + D_v \nabla^2 v_1 \\
\frac{\partial u_2}{\partial t} &= b_2 v_2 + \gamma_2 \frac{K_1^n}{K_1^n + u_1^n} v_2 - \delta u_2 + D_u \nabla^2 u_2 \\
\frac{\partial v_2}{\partial t} &= -b_2 v_2 - \gamma_2 \frac{K_1^n}{K_1^n + u_1^n} v_2 + \delta u_2 + D_v \nabla^2 v_2,
\end{aligned} \tag{8}$$

where u_i and v_i are the concentrations of active and inactive GTPase i respectively, γ_i is the activation rate of GTPase i that can be inhibited by the other GTPase, K_i is the active GTPase i concentration at which the γ of the other GTPase is halved. Other parameters are as in the WP model. For convenience, inactivation rate constant δ is assumed equal for both GTPases. The average concentrations T_1 and T_2 of GTPase$_1$ and GTPase$_2$ are as in Eq 2. The dimensionless variables are:

$$\tilde{u}_1 = \frac{u_1}{K_1}, \quad \tilde{u}_2 = \frac{u_2}{K_2}, \quad \tilde{v}_1 = \frac{v_1}{K_1}, \quad \tilde{v}_2 = \frac{v_2}{K_2}, \quad \tilde{t} = \delta t, \quad \tilde{x} = \frac{x\sqrt{\delta}}{\sqrt{D_u}}, \quad \tilde{y} = \frac{y\sqrt{\delta}}{\sqrt{D_u}}, \tag{9}$$

and the scaled parameters:

$$\begin{aligned}
&\tilde{T}_1 = \frac{T_1}{K_1}, \quad \tilde{T}_2 = \frac{T_2}{K_2}, \quad \tilde{b}_1 = \frac{b_1}{\delta}, \quad \tilde{b}_2 = \frac{b_2}{\delta}, \quad \tilde{\gamma}_1 = \frac{\gamma_1}{\delta}, \quad \tilde{\gamma}_2 = \frac{\gamma_2}{\delta}, \\
&\tilde{D}_v = \frac{D_v}{D_u}, \quad \tilde{A} = A\frac{\delta}{D_u}.
\end{aligned} \tag{10}$$

This results in a dimensionless total amount of GTPase as in Eq 5. Dropping the tildes, we obtain the dimensionless MI model:

$$\begin{aligned}
\frac{\partial u_1}{\partial t} &= b_1 v_1 + \gamma_1 \frac{1}{1 + u_2^n} v_1 - u_1 + \nabla^2 u_1 \\
\frac{\partial v_1}{\partial t} &= -b_1 v_1 - \gamma_1 \frac{1}{1 + u_2^n} v_1 + u_1 + D_v \nabla^2 v_1 \\
\frac{\partial u_2}{\partial t} &= b_2 v_2 + \gamma_2 \frac{1}{1 + u_1^n} v_2 - u_2 + \nabla^2 u_2 \\
\frac{\partial v_2}{\partial t} &= -b_2 v_2 - \gamma_2 \frac{1}{1 + u_1^n} v_2 + u_2 + D_v \nabla^2 v_2.
\end{aligned} \tag{11}$$

1.3 Non-dimensionalisation of the WPT model

The full dimension-carrying WPT model is given by:

$$\begin{aligned}
\frac{\partial u}{\partial t} &= bv + \gamma \frac{u^n}{K^n + u^n} v - \delta u - \xi u + D_u \nabla^2 u \\
\frac{\partial v}{\partial t} &= -bv - \gamma \frac{u^n}{K^n + u^n} v + \delta u + \sigma + D_v \nabla^2 v,
\end{aligned} \tag{12}$$

where ξ is the degradation rate of active GTPase and σ is a constant production term for inactive GTPase. Other symbols are as in previous models. The dimensionless variables are:

$$\tilde{u} = \frac{u}{K}, \quad \tilde{v} = \frac{v}{K}, \quad \tilde{t} = \delta t, \quad \tilde{x} = \frac{x\sqrt{\delta}}{\sqrt{D_u}}, \quad \tilde{y} = \frac{y\sqrt{\delta}}{\sqrt{D_u}}, \tag{13}$$

and the scaled parameters:

$$\tilde{b} = \frac{b}{\delta}, \quad \tilde{\gamma} = \frac{\gamma}{\delta}, \quad \tilde{\xi} = \frac{\xi}{\delta}, \quad \tilde{\sigma} = \frac{\sigma}{\delta K}, \quad \tilde{D}_v = \frac{D_v}{D_u}. \tag{14}$$

Dropping the tildes, we obtain the dimensionless WPT model:

$$\begin{aligned} \frac{\partial u}{\partial t} &= bv + \gamma \frac{u^n}{1+u^n} v - u - \xi u + \nabla^2 u \\ \frac{\partial v}{\partial t} &= -bv - \gamma \frac{u^n}{1+u^n} v + u + \sigma + D_v \nabla^2 v. \end{aligned} \tag{15}$$

1.4 Non-dimensionalisation of the MIT model

The full dimension-carrying system is given by:

$$\begin{aligned} \frac{\partial u_1}{\partial t} &= b_1 v_1 + \gamma_1 \frac{K_2^n}{K_2^n + u_2^n} v_1 - \delta u_1 - \xi_1 u_1 + D_u \nabla^2 u_1 \\ \frac{\partial v_1}{\partial t} &= -b_1 v_1 - \gamma_1 \frac{K_2^n}{K_2^n + u_2^n} v_1 + \delta u_1 + \sigma_1 + D_v \nabla^2 v_1 \\ \frac{\partial u_2}{\partial t} &= b_2 v_2 + \gamma_2 \frac{K_1^n}{K_1^n + u_1^n} v_2 - \delta u_2 - \xi_2 u_2 + D_u \nabla^2 u_2 \\ \frac{\partial v_2}{\partial t} &= -b_2 v_2 - \gamma_2 \frac{K_1^n}{K_1^n + u_1^n} v_2 + \delta u_2 + \sigma_2 + D_v \nabla^2 v_2, \end{aligned} \tag{16}$$

with variables and parameters as in the previous models. The dimensionless variables are:

$$\tilde{u}_1 = \frac{u_1}{K_1}, \quad \tilde{u}_2 = \frac{u_2}{K_2}, \quad \tilde{v}_1 = \frac{v_1}{K_1}, \quad \tilde{v}_2 = \frac{v_2}{K_2}, \quad \tilde{t} = \delta t, \quad \tilde{x} = \frac{x\sqrt{\delta}}{\sqrt{D_u}}, \quad \tilde{y} = \frac{y\sqrt{\delta}}{\sqrt{D_u}}, \tag{17}$$

and the scaled parameters:

$$\begin{aligned} &\tilde{b}_1 = \frac{b_1}{\delta}, \quad \tilde{b}_2 = \frac{b_2}{\delta}, \quad \tilde{\gamma}_1 = \frac{\gamma_1}{\delta}, \quad \tilde{\gamma}_2 = \frac{\gamma_2}{\delta}, \quad \tilde{\xi}_1 = \frac{\xi_1}{\delta}, \quad \tilde{\xi}_2 = \frac{\xi_2}{\delta}, \\ &\tilde{\sigma}_1 = \frac{\sigma_1}{\delta K_1}, \quad \tilde{\sigma}_2 = \frac{\sigma_2}{\delta K_2}, \quad \tilde{D}_v = \frac{D_v}{D_u}. \end{aligned} \tag{18}$$

Dropping the tildes, we obtain the dimensionless MIT model:

$$\begin{aligned} \frac{\partial u_1}{\partial t} &= b_1 v_1 + \gamma_1 \frac{1}{1+u_2^n} v_1 - u_1 - \xi_1 u_1 + \nabla^2 u_1 \\ \frac{\partial v_1}{\partial t} &= -b_1 v_1 - \gamma_1 \frac{1}{1+u_2^n} v_1 + u_1 + \sigma_1 + D_v \nabla^2 v_1 \\ \frac{\partial u_2}{\partial t} &= b_2 v_2 + \gamma_2 \frac{1}{1+u_1^n} v_2 - u_2 - \xi_2 u_2 + \nabla^2 u_2 \\ \frac{\partial v_2}{\partial t} &= -b_2 v_2 - \gamma_2 \frac{1}{1+u_1^n} v_2 + u_2 + \sigma_2 + D_v \nabla^2 v_2. \end{aligned} \tag{19}$$

1.5 Non-dimensionalisation of the WPGAP model

The full dimension-carrying WPGAP model is given by:

$$
\begin{aligned}
\frac{\partial u}{\partial t} &= bv + \gamma \frac{u^n}{K^n + u^n} v - \delta u - eGu + D_u \nabla^2 u \\
\frac{\partial v}{\partial t} &= -bv - \gamma \frac{u^n}{K^n + u^n} v + \delta u + eGu + D_v \nabla^2 v \\
\frac{\partial G}{\partial t} &= cug - dG + D_G \nabla^2 G \\
\frac{\partial g}{\partial t} &= -cug + dG + D_g \nabla^2 g,
\end{aligned}
\tag{20}
$$

where G and g are the concentrations of active and inactive GAP respectively, D_G and D_g are the diffusion coefficients of active and inactive GAP respectively, c is a GTPase dependent GAP activation rate constant, d is the GAP inactivation rate constant, and e is a GAP dependent GTPase inactivation constant. This model has conservation parameter T as in Eq 2 and an average total GAP concentration:

$$
T_g = \frac{1}{A} \iint_A (G + g) dx dy = \frac{\text{Total GAP}}{A}. \tag{21}
$$

The dimensionless variables are:

$$
\tilde{u} = \frac{u}{K}, \quad \tilde{v} = \frac{v}{K}, \quad \tilde{G} = \frac{e}{\delta} G, \quad \tilde{g} = \frac{e}{\delta} g, \quad \tilde{t} = \delta t, \quad \tilde{x} = \frac{x\sqrt{\delta}}{\sqrt{D_u}}, \quad \tilde{y} = \frac{y\sqrt{\delta}}{\sqrt{D_u}}, \tag{22}
$$

and the scaled parameters:

$$
\begin{aligned}
&\tilde{T} = \frac{T}{K}, \quad \tilde{T}_g = \frac{eT_g}{\delta}, \quad \tilde{b} = \frac{b}{\delta}, \quad \tilde{\gamma} = \frac{\gamma}{\delta}, \quad \tilde{c} = \frac{cK}{\delta}, \quad \tilde{d} = \frac{d}{\delta}, \\
&\tilde{D}_v = \frac{D_v}{D_u}, \quad \tilde{D}_G = \frac{D_G}{D_u}, \quad \tilde{D}_g = \frac{D_g}{D_u}, \quad \tilde{A} = A \frac{\delta}{D_u}.
\end{aligned}
\tag{23}
$$

This makes the dimensionless total amount of GTPase:

$$
\tilde{\text{Total GAP}} = \frac{\text{Total GAP} \cdot e}{D_u}. \tag{24}
$$

Dropping the tildes, we obtain the dimensionless WPGAP model:

$$
\begin{aligned}
\frac{\partial u}{\partial t} &= bv + \gamma \frac{u^n}{1 + u^n} v - u - Gu + D_u \nabla^2 u \\
\frac{\partial v}{\partial t} &= -bv - \gamma \frac{u^n}{1 + u^n} v + u + Gu + D_v \nabla^2 v \\
\frac{\partial G}{\partial t} &= cug - dG + D_G \nabla^2 G \\
\frac{\partial g}{\partial t} &= -cug + dG + D_g \nabla^2 g,
\end{aligned}
\tag{25}
$$

with a dimensionless total average GAP concentration:

$$
T_g = \frac{1}{A} \iint_A (G + g) dx dy = \frac{\text{Total GAP}}{A}. \tag{26}
$$

1.6 Non-dimensionalisation of the MIGAP1 model

The full dimension-carrying system is given by:

$$\begin{aligned}
\frac{\partial u_1}{\partial t} &= b_1 v_1 + \gamma_1 \frac{K_2^n}{K_2^n + u_2^n} v_1 - \delta u_1 - e_1 G_1 u_1 + D_u \nabla^2 u_1 \\
\frac{\partial v_1}{\partial t} &= -b_1 v_1 - \gamma_1 \frac{K_2^n}{K_2^n + u_2^n} v_1 + \delta u_1 + e_1 G_1 u_1 + D_v \nabla^2 v_1 \\
\frac{\partial u_2}{\partial t} &= b_2 v_2 + \gamma_2 \frac{K_1^n}{K_1^n + u_1^n} v_2 - \delta u_2 + D_u \nabla^2 u_2 \\
\frac{\partial v_2}{\partial t} &= -b_2 v_2 - \gamma_2 \frac{K_1^n}{K_1^n + u_1^n} v_2 + \delta u_2 + D_v \nabla^2 v_2 \\
\frac{\partial G_1}{\partial t} &= c_1 u_1 g_1 - d_1 G_1 + D_G \nabla^2 G_1 \\
\frac{\partial g_1}{\partial t} &= -c_1 u_1 g_1 + d_1 G_1 + D_g \nabla^2 g_1,
\end{aligned} \tag{27}$$

with variables and parameters as in previous models. This system has two average total GTPase concentrations as in Eq 2 and one average total GAP concentration as in Eq 21. The dimensionless variables are:

$$\begin{aligned}
&\tilde{u}_1 = \frac{u_1}{K_1}, \quad \tilde{u}_2 = \frac{u_2}{K_2}, \quad \tilde{v}_1 = \frac{v_1}{K_1}, \quad \tilde{v}_2 = \frac{v_2}{K_2}, \quad \tilde{G}_1 = \frac{e_1}{\delta} G_1, \quad \tilde{g}_1 = \frac{e_1}{\delta} g_1, \\
&\tilde{t} = \delta t, \quad \tilde{x} = \frac{x\sqrt{\delta}}{\sqrt{D_u}}, \quad \tilde{y} = \frac{y\sqrt{\delta}}{\sqrt{D_u}},
\end{aligned} \tag{28}$$

and the scaled parameters:

$$\begin{aligned}
&\tilde{T}_1 = \frac{T_1}{K_1}, \quad \tilde{T}_2 = \frac{T_2}{K_2}, \quad \tilde{T}_{g,1} = \frac{e_1 T_{g,1}}{\delta}, \quad \tilde{b}_1 = \frac{b_1}{\delta}, \quad \tilde{b}_2 = \frac{b_2}{\delta}, \quad \tilde{\gamma}_1 = \frac{\gamma_1}{\delta}, \quad \tilde{\gamma}_2 = \frac{\gamma_2}{\delta}, \\
&\tilde{c}_1 = \frac{c_1 K_1}{\delta}, \quad \tilde{d}_1 = \frac{d_1}{\delta}, \quad \tilde{D}_v = \frac{D_v}{D_u}, \quad \tilde{D}_G = \frac{D_G}{D_u}, \quad \tilde{D}_g = \frac{D_g}{D_u}, \quad \tilde{A} = A\frac{\delta}{D_u}.
\end{aligned} \tag{29}$$

Dropping the tildes, we obtain the dimensionless MIGAP1 model:

$$\begin{aligned}
\frac{\partial u_1}{\partial t} &= b_1 v_1 + \gamma_1 \frac{1}{1 + u_2^n} v_1 - u_1 - G_1 u_1 + \nabla^2 u_1 \\
\frac{\partial v_1}{\partial t} &= -b_1 v_1 - \gamma_1 \frac{1}{1 + u_2^n} v_1 + u_1 + G_1 u_1 + D_v \nabla^2 v_1 \\
\frac{\partial u_2}{\partial t} &= b_2 v_2 + \gamma_2 \frac{1}{1 + u_1^n} v_2 - u_2 + \nabla^2 u_2 \\
\frac{\partial v_2}{\partial t} &= -b_2 v_2 - \gamma_2 \frac{1}{1 + u_1^n} v_2 + u_2 + D_v \nabla^2 v_2 \\
\frac{\partial G_1}{\partial t} &= c_1 u_1 g_1 - d_1 G_1 + D_G \nabla^2 G_1 \\
\frac{\partial g_1}{\partial t} &= -c_1 u_1 g_1 + d_1 G_1 + D_g \nabla^2 g_1.
\end{aligned} \tag{30}$$

1.7 Non-dimensionalisation of the MIGAP2 model

The full dimension-carrying MIGAP2 system is given by:

$$\begin{aligned}
\frac{\partial u_1}{\partial t} &= b_1 v_1 + \gamma_1 \frac{K_2^n}{K_2^n + u_2^n} v_1 - \delta u_1 - e_1 G_1 u_1 + D_u \nabla^2 u_1 \\
\frac{\partial v_1}{\partial t} &= -b_1 v_1 - \gamma_1 \frac{K_2^n}{K_2^n + u_2^n} v_1 + \delta u_1 + e_1 G_1 u_1 + D_v \nabla^2 v_1 \\
\frac{\partial u_2}{\partial t} &= b_2 v_2 + \gamma_2 \frac{K_1^n}{K_1^n + u_1^n} v_2 - \delta u_2 - e_2 G_2 u_2 + D_u \nabla^2 u_2 \\
\frac{\partial v_2}{\partial t} &= -b_2 v_2 - \gamma_2 \frac{K_1^n}{K_1^n + u_1^n} v_2 + \delta u_2 + e_2 G_2 u_2 + D_v \nabla^2 v_2 \\
\frac{\partial G_1}{\partial t} &= c_1 u_1 g_1 - d_1 G_1 + D_G \nabla^2 G_1 \\
\frac{\partial g_1}{\partial t} &= -c_1 u_1 g_1 + d_1 G_1 + D_g \nabla^2 g_1 \\
\frac{\partial G_2}{\partial t} &= c_2 u_2 g_2 - d_2 G_2 + D_G \nabla^2 G_2 \\
\frac{\partial g_2}{\partial t} &= -c_2 u_2 g_2 + d_2 G_2 + D_g \nabla^2 g_2,
\end{aligned} \tag{31}$$

with variables and parameters as in previous models. This model has two average total GTPase concentrations as in Eq 2 and two average total GAP concentrations as in Eq 21. The dimensionless variables are:

$$\begin{aligned}
&\tilde{u}_1 = \frac{u_1}{K_1}, \quad \tilde{u}_2 = \frac{u_2}{K_2}, \quad \tilde{v}_1 = \frac{v_1}{K_1}, \quad \tilde{v}_2 = \frac{v_2}{K_2}, \\
&\tilde{G}_1 = \frac{e_1}{\delta} G_1, \quad \tilde{g}_1 = \frac{e_1}{\delta} g_1, \quad \tilde{G}_2 = \frac{e_2}{\delta} G_2, \quad \tilde{g}_2 = \frac{e_2}{\delta} g_2, \quad \tilde{t} = \delta t, \quad \tilde{x} = \frac{x\sqrt{\delta}}{\sqrt{D_u}}, \quad \tilde{y} = \frac{y\sqrt{\delta}}{\sqrt{D_u}},
\end{aligned} \tag{32}$$

and the scaled parameters:

$$\begin{aligned}
&\tilde{T}_1 = \frac{T_1}{K_1}, \quad \tilde{T}_2 = \frac{T_2}{K_2}, \quad \tilde{T}_{g,1} = \frac{e_1 T_{g,1}}{\delta}, \quad \tilde{T}_{g,2} = \frac{e_2 T_{g,2}}{\delta}, \quad \tilde{b}_1 = \frac{b_1}{\delta}, \quad \tilde{b}_2 = \frac{b_2}{\delta}, \\
&\tilde{\gamma}_1 = \frac{\gamma_1}{\delta}, \quad \tilde{\gamma}_2 = \frac{\gamma_2}{\delta}, \quad \tilde{c}_1 = \frac{c_1 K_1}{\delta}, \quad \tilde{d}_1 = \frac{d_1}{\delta}, \quad \tilde{c}_2 = \frac{c_2 K_2}{\delta}, \quad \tilde{d}_2 = \frac{d_2}{\delta}, \\
&\tilde{D}_v = \frac{D_v}{D_u}, \quad \tilde{D}_G = \frac{D_G}{D_u}, \quad \tilde{D}_g = \frac{D_g}{D_u}, \quad \tilde{A} = A\frac{\delta}{D_u}.
\end{aligned} \tag{33}$$

Dropping the tildes, we obtain the dimensionless MIGAP2 model:

$$\begin{aligned}
\frac{\partial u_1}{\partial t} &= b_1 v_1 + \gamma_1 \frac{1}{1+u_2^n} v_1 - u_1 - G_1 u_1 + \nabla^2 u_1 \\
\frac{\partial v_1}{\partial t} &= -b_1 v_1 - \gamma_1 \frac{1}{1+u_2^n} v_1 + u_1 + G_1 u_1 + D_v \nabla^2 v_1 \\
\frac{\partial u_2}{\partial t} &= b_2 v_2 + \gamma_2 \frac{1}{1+u_1^n} v_2 - u_2 - G_2 u_2 + \nabla^2 u_2 \\
\frac{\partial v_2}{\partial t} &= -b_2 v_2 - \gamma_2 \frac{1}{1+u_1^n} v_2 + u_2 + G_2 u_2 + D_v \nabla^2 v_2 \\
\frac{\partial G_1}{\partial t} &= c_1 u_1 g_1 - d_1 G_1 + D_G \nabla^2 G_1 \\
\frac{\partial g_1}{\partial t} &= -c_1 u_1 g_1 + d_1 G_1 + D_g \nabla^2 g_1 \\
\frac{\partial G_2}{\partial t} &= c_2 u_2 g_2 - d_2 G_2 + D_G \nabla^2 G_2 \\
\frac{\partial g_2}{\partial t} &= -c_2 u_2 g_2 + d_2 G_2 + D_g \nabla^2 g_2 .
\end{aligned} \tag{34}$$

2 Homogeneous steady states

The WP model has the following homogeneous steady state (HSS):

$$\begin{aligned}
u_{HSS} &= u_0 \\
v_{HSS} &= \frac{u_0(1+u_0^n)}{(b+\gamma)u_0^n + b},
\end{aligned} \tag{35}$$

where u_0 is the concentration of u at the homogeneous steady state for a specific average GTPase concentration $T = u_{HSS} + v_{HSS}$.

The MI model has the following homogeneous steady state:

$$\begin{aligned}
u_{1,HSS} &= u_{1,0} \\
v_{1,HSS} &= u_{1,0} \frac{1 + \left(T_2 \frac{b_2 u_{1,0}^n + b_2 + \gamma_2}{1 + b_2 u_{1,0}^n + u_{1,0}^n + b_2 + \gamma_2} \right)^n}{b_1 + \gamma_1 + b_1 \left(T_2 \frac{b_2 u_{1,0}^n + b_2 + \gamma_2}{1 + b_2 u_{1,0}^n + u_{1,0}^n + b_2 + \gamma_2} \right)^n} \\
u_{2,HSS} &= T_2 \frac{b_2 u_{1,0}^n + b_2 + \gamma_2}{b_2 u_{1,0}^n + u_{1,0}^n + b_2 + \gamma_2 + 1} \\
v_{2,HSS} &= T_2 - u_{2,HSS},
\end{aligned} \tag{36}$$

where $u_{1,0}$ is the concentration of u at the homogeneous steady state for a specific average GTPase 1 concentration $T_1 = u_{1,HSS} + v_{1,HSS}$. For the second GTPase, the average concentration $T_2 = u_{2,HSS} + v_{2,HSS}$ was used as an extra parameter.

For the WPT model, the homogeneous steady state is:

$$\begin{aligned} u_{HSS} &= \frac{\sigma}{\xi} \\ v_{HSS} &= \frac{\sigma\left(\left(\frac{\sigma}{\xi}\right)^n + 1\right)(\xi+1)}{\xi\left((b+\gamma)\left(\frac{\sigma}{\xi}\right)^n + b\right)}. \end{aligned} \tag{37}$$

The MIT model has the following homogeneous steady state:

$$\begin{aligned} u_{1,HSS} &= \frac{\sigma_1}{\xi_1} \\ v_{1,HSS} &= \frac{\sigma_1\left(\left(\frac{\sigma_2}{\xi_2}\right)^n + 1\right)(\xi_1+1)}{\xi_1\left(b_1\left(\frac{\sigma_2}{\xi_2}\right)^n + b_1 + \gamma_1\right)} \\ u_{2,HSS} &= \frac{\sigma_2}{\xi_2} \\ v_{2,HSS} &= \frac{\sigma_2\left(\left(\frac{\sigma_1}{\xi_1}\right)^n + 1\right)(\xi_2+1)}{\xi_2\left(b_2\left(\frac{\sigma_1}{\xi_1}\right)^n + b_2 + \gamma_2\right)}. \end{aligned} \tag{38}$$

The homogeneous steady state of the WPGAP model is:

$$\begin{aligned} u_{HSS} &= u_0 \\ v_{HSS} &= u_0 \frac{c(T_g+1)u_0^{n+1} + d\cdot u_0^n + c\cdot u_0(T_g+1) + d}{\left((b+\gamma)u_0^n + b\right)(c\cdot u_0 + d)} \\ G_{HSS} &= \frac{c\cdot u_0\cdot T_g}{c\cdot u_0 + d} \\ g_{HSS} &= T_g - G_{HSS}, \end{aligned} \tag{39}$$

where $T_g = G_{HSS} + g_{HSS}$ is the average concentration of GAP.

The homogeneous steady state of the MIGAP1 model is:

$$\begin{aligned} u_{1,HSS} &= u_{1,0} \\ v_{1,HSS} &= u_{1,0} \frac{\left(1 + \left(T_2 \frac{b_2 u_{1,0}^n + b_2 + \gamma_2}{1 + b_2 u_{1,0}^n + u_{1,0}^n + b_2 + \gamma_2}\right)^n\right)(c(T_g+1)u_{1,0} + d)}{\left(b_1 + \gamma_1 + b_1\left(T_2 \frac{b_2 u_{1,0}^n + b2 + \gamma_2}{1 + b_2 u_{1,0}^n + u_{1,0}^n + b_2 + \gamma_2}\right)^n\right)(c\cdot u_{1,0} + d)} \\ u_{2,HSS} &= T_2 \frac{b_2 u_{1,0}^n + b_2 + \gamma_2}{b_2 u_{1,0}^n + u_{1,0}^n + b_2 + \gamma_2 + 1} \\ v_{2,HSS} &= T_2 - u_{2,HSS} \\ G_{1,HSS} &= \frac{c\cdot u_{1,0}\cdot T_g}{c\cdot u_{1,0} + d} \\ g_{1,HSS} &= T_g - G_{1,HSS}, \end{aligned} \tag{40}$$

The MIGAP2 model has the following homogeneous steady state:

$$
\begin{aligned}
u_{1,HSS} &= u_{1,0} \\
v_{1,HSS} &= u_{1,0}\frac{(1+u_{2,HSS}^n)(c_1(T_{g,1}+1)u_{1,0}+d_1)}{(b_1u_{2,HSS}^n+b_1+\gamma_1)(c_1u_{1,0}+d_1)} \\
u_{2,HSS} &= \frac{-B\pm\sqrt{B^2-4AC}}{2A} \\
A &= (c_2T_{g,2}+b_2c_2+c_2)u_{1,0}^n+c_2T_{g,2}+b_2c_2+c_2\gamma_2+c_2 \\
B &= (b_2d_2+d_2-c_2b_2T_2)u_{1,0}^n+b_2d_2+d_2\gamma_2+d_2-c_2b_2T_2-c_2\gamma_2T_2 \\
C &= -d_2T_2(u_{1,0}^nb_2+b_2+\gamma_2) \\
v_{2,HSS} &= T_2-u_{2,HSS} \\
G_{1,HSS} &= \frac{c_1\cdot u_{1,0}\cdot T_{g,1}}{c_1\cdot u_{1,0}+d_1} \\
g_{1,HSS} &= T_{g,1}-G_{1,HSS} \\
G_{2,HSS} &= \frac{c_2\cdot u_{2,0}\cdot T_{g,2}}{c_2\cdot u_{2,0}+d_2} \\
g_{2,HSS} &= T_{g,2}-G_{2,HSS}.
\end{aligned} \tag{41}
$$

There are two possibilities for $u_{2,HSS}$, resulting as solutions from a quadratic equation. However, since the term $-4AC$ is always positive, there is always exactly one positive option, which we used for the simulation.

3 Linear stability analysis for two-component reaction-diffusion systems in two dimensions

3.1 Linearisation and growth modes

Linear stability analysis (LSA) can be used to determine under what conditions arbitrarily small spatial perturbations in a homogeneous state can grow, leading to a heterogeneous state. LSA was performed as previously described (Murray, 2001) for reaction-diffusion systems of the following (dimensionless) structure:

$$
\begin{aligned}
\frac{\partial u}{\partial t} &= \nabla^2 u + f(u,v) \\
\frac{\partial v}{\partial t} &= D_v\nabla^2 v + g(u,v),
\end{aligned} \tag{42}
$$

where u is the concentration of the slow-diffusing activator, v is the concentration of the fast-diffusing substrate, D_v the diffusion coefficient of v relative to that of u, ∇^2 the two-dimensional Laplace operator, and f and g are (non-linear) functions describing the interaction between u and v. Linearisation of this system about the homogeneous steady state gives (in matrix-vector form):

$$
\begin{aligned}
\frac{\partial w}{\partial t} &= D\nabla^2 w + Jw \\
w &= \begin{bmatrix}\Delta u\\ \Delta v\end{bmatrix} \quad D=\begin{bmatrix}1 & 0\\ 0 & D_v\end{bmatrix} \quad J=\begin{bmatrix}f_u & f_v\\ g_u & g_v\end{bmatrix} \\
\Delta u &= u-u_{HSS} \quad \Delta v = v-v_{HSS},
\end{aligned} \tag{43}
$$

where u_{HSS} and v_{HSS} are the concentrations of u and v at the homogeneous steady state respectively, and J is the Jacobian matrix with partial derivatives of f and g with respect to u and v evaluated at the homogeneous steady state. This linear system has solutions of the form:

$$w(x, y, t) = \sum_k c_k e^{\lambda_k t} W_k(x, y). \tag{44}$$

Here, c_k is a constant (determined by initial conditions) belonging to wave number k, λ_k is a the growth mode belonging to wave number k and $W_k(x, y)$ is the time-independent solution of the following eigenvalue problem:

$$\nabla^2 W(x, y) = -k^2 W(x, y). \tag{45}$$

On finite domains only discrete wave numbers are admissible. Which wave numbers are allowed is determined by the size of the domain and the boundary conditions.

The growth modes λ_k as a function of the wave number can be determined by substituting Eq 44 and Eq 45 into Eq 43:

$$\begin{aligned} \lambda w &= -k^2 D w + J w \\ (J - k^2 D) w &= \lambda w. \end{aligned} \tag{46}$$

These growth modes are then given by the roots of the following equation:

$$\det\big(J - k^2 D - \lambda_k I\big) = 0. \tag{47}$$

This leads to the following solutions for λ_k:

$$\begin{aligned} \lambda_k = &-\frac{1}{2}k^2(1 + D_v) + \frac{1}{2}(f_u + g_v) \pm \\ &\frac{1}{2}\sqrt{(1 - D_v)^2 k^4 - 2(f_u - g_v)(1 - D_v)k^2 + f_u^2 - 2f_u g_v + 4f_v g_u + g_v^2}. \end{aligned} \tag{48}$$

If the real part of any λ_k belonging to an admissible wave number is greater than zero, the homogeneous steady state is unstable, small perturbations will drive spontaneous pattern formation.

3.2 Wave numbers

Admissible wave numbers can be determined by solving Eq 45. To this end we split $W_k(x, y)$ into an x-dependent and a y-dependent part:

$$W_k(x, y) = \bar{v} X(x) Y(y), \tag{49}$$

where $X(x)$ is a scalar function of x, $Y(y)$ is a scalar function of y, and $\bar{v}$ is a constant vector containing the coefficients for components u and v. Substituting this into Eq 45 results in:

$$\bar{v} X''(x) Y(y) + \bar{v} X(x) Y''(y) = -k^2 \bar{v} X(x) Y(y). \tag{50}$$

Division by $X(x)Y(y)$ gives:

$$\left(\frac{X''(x)}{X(x)} + \frac{Y''(y)}{Y(y)} \right) \bar{v} = -k^2 \bar{v}. \tag{51}$$

This equation can be separated into an x-dependent and a y-dependent part that both need to be constant:

$$\begin{aligned} \frac{X''(x)}{X(x)} &= a \\ \frac{Y''(y)}{Y(y)} &= b \\ a + b &= -k^2, \end{aligned} \tag{52}$$

where a and b are constants to be solved from their respective one-dimensional boundary value problems. The results depend on the boundary conditions and the domain size. We will consider a rectangular domain with periodic boundary conditions in the x-direction and homogeneous Neumann boundary conditions in the y-direction. This results in a geometry resembling an open cylinder.

3.2.1 Periodic boundary conditions

The periodic boundary conditions in the x-direction result in the following boundary value problem:

$$\begin{aligned} X''(x) &= aX(x) \\ X(0) &= X(L) \\ X'(0) &= X'(L), \end{aligned} \tag{53}$$

where L is the length of the domain in the x-direction (or the circumference of the open cylinder). This problem only has trivial solutions for $a > 0$. For $a = 0$, there is a single solution where $X(x)$ is constant. For $a < 0$, with $a = -\mu^2$, solutions follow:

$$\begin{aligned} X(x) &= A\cos(\mu x) + B\sin(\mu x) \\ X'(x) &= -A\mu\sin(\mu x) + B\mu\cos(\mu x), \end{aligned} \tag{54}$$

where A and B are constants. Substituting these equations into the boundary conditions results in:

$$\begin{aligned} A\sin(\mu L) + B(1 - \cos(\mu L)) &= 0 \\ A(1 - \cos(\mu L)) - B\sin(\mu L) &= 0, \end{aligned} \tag{55}$$

which can be rewritten in matrix-vector form:

$$\begin{bmatrix} \sin(\mu L) & 1 - \cos(\mu L) \\ 1 - \cos(\mu L) & -\sin(\mu L) \end{bmatrix} \begin{bmatrix} A \\ B \end{bmatrix} = \begin{bmatrix} 0 \\ 0 \end{bmatrix}. \tag{56}$$

For non-trivial solutions, the determinant of the matrix should be zero, so:

$$\begin{aligned} & -\sin^2(\mu L) - (1 - \cos(\mu L))^2 = 0 \\ & \sin^2(\mu L) + 1 - 2\cos(\mu L) + \cos^2(\mu L) = 0 \\ & 2 - 2\cos(\mu L) = 0 \\ & \cos(\mu L) = 1 \\ & \mu_n = \frac{2n\pi}{L}, \quad n \in \mathbb{N}. \end{aligned} \tag{57}$$

Therefore, admissible values of a with periodic boundary conditions in the x-direction are:

$$a_n = -\mu_n^2 = -\frac{4n^2\pi^2}{L^2}, \quad n \in \mathbb{N}. \tag{58}$$

3.2.2 Zero flux boundary conditions

The zero flux boundary conditions in the y-direction determine the following boundary value problem:

$$\begin{aligned} Y''(y) &= bY(y) \\ Y'(0) &= 0 \\ Y'(H) &= 0, \end{aligned} \tag{59}$$

where H is the length of the domain in the y-direction (the height of the open cylinder). For $b > 0$ only trivial solutions exist. For $b = 0$, there is again a single solution where $Y(y)$ is constant. For $b < 0$, with $b = -\nu^2$, solutions follow:

$$\begin{aligned} Y(y) &= A\cos(\nu y) + B\sin(\nu y) \\ Y'(y) &= -A\nu\sin(\nu y) + B\nu\cos(\nu y), \end{aligned} \tag{60}$$

where A and B are constants. Substitution into the boundary conditions yields:

$$\begin{aligned} Y'(0) = \quad & \nu B = 0 \\ & B = 0 \\ Y'(H) = \quad & -\nu A\sin(\nu H) = 0 \\ & \sin(\nu H) = 0 \\ & \nu_m = \frac{m\pi}{H}, \quad m \in \mathbb{N}. \end{aligned} \tag{61}$$

Therefore, with zero flux boundary conditions in the y-direction, the following values of b are admissible:

$$b_m = -\nu_m^2 = -\frac{m^2\pi^2}{H^2}, \quad m \in \mathbb{N}. \tag{62}$$

3.2.3 Admissible wave numbers

Combining a and b, the admissible wave numbers for periodic boundary conditions in the x-direction and zero flux boundary conditions in the y-direction are given by:

$$k_{n,m}^2 = -(a_n + b_m) = \pi^2\left(\frac{4n^2}{L^2} + \frac{m^2}{H^2}\right). \tag{63}$$

3.3 One parameter bifurcation analysis

Provided at least one admissible wave number exists, the homogeneous steady state becomes unstable when $\max(\mathrm{Re}(\lambda_k)) > 0$, with λ_k as given by Eq 48. To determine how $\max(\mathrm{Re}(\lambda_k))$ depends on the parameters, the values of k^2 for which $\mathrm{Re}(\lambda_k)$ is maximal can be determined as previously described (Miura and Maini, 2004). There are two branches of λ_k:

$$\begin{aligned} \lambda_{k+} &= E + \frac{1}{2}\sqrt{F} \\ \lambda_{k-} &= E - \frac{1}{2}\sqrt{F} \\ E &= -\frac{1}{2}k^2(1 + D_v) + \frac{1}{2}(f_u + g_v) \\ F &= (1 - D_v)^2k^4 - 2(f_u - g_v)(1 - D_v)k^2 + f_u^2 - 2f_ug_v + 4f_vg_u + g_v^2. \end{aligned} \tag{64}$$

Since $\mathrm{Re}(\lambda_{k+}) \geq \mathrm{Re}(\lambda_{k-})$, only λ_{k+} needs to be considered. To find the maximum we take the derivative of this branch of $\lambda_k$to k^2 and set it equal to zero:

$$\begin{aligned}\frac{d\lambda_{k+}}{dk^2} &= -\frac{1}{2}(1+D_v)+ \\ &\frac{1}{2}\frac{(1-D_v)^2k^2-(f_u-g_v)(1-D_v)}{\sqrt{(1-D_v)^2k^4-2(f_u-g_v)(1-D_v)k^2+f_u^2-2f_ug_v+4f_vg_u+g_v^2}} \\ &= 0.\end{aligned} \tag{65}$$

Solving this equation results in the following relation for k^2:

$$k^2 = \frac{-(f_u-g_v)D_v \pm (1+D_v)\sqrt{-D_vf_vg_u}}{D_v(D_v-1)}. \tag{66}$$

Since $D_v > 1$ and $f_u - g_v > 0$ for substrate depletion models, and only positive values of k^2 are relevant (wave numbers should be real), this relation can be reduced to:

$$k^2 = \frac{-(f_u-g_v)D_v + (1+D_v)\sqrt{-D_vf_vg_u}}{D_v(D_v-1)}. \tag{67}$$

Using Eq 67 and Eq 48, the maximum value of λ_k can be determined as a function of model parameters if the Jacobian matrix of the system at the homogeneous steady state is known. Homogeneous steady states are given in section 2. For the WP model the Jacobian matrix at the homogeneous steady state is given by:

$$J = \begin{bmatrix} \dfrac{((n-2)\gamma-3b)u_0^{2n}+(-b-\gamma)u_0^{3n}+((n-1)\gamma-3b)u_0^n-b}{((b+\gamma)u_0^n+b)(1+u_0^n)^2} & b+\dfrac{\gamma u_0^n}{1+u_0^n} \\ \dfrac{((-n+2)\gamma+3b)u_0^{2n}+(b+\gamma)u_0^{3n}+((-n+1)\gamma+3b)u_0^n+b}{((b+\gamma)u_0^n+b)(1+u_0^n)^2} & -b-\dfrac{\gamma u_0^n}{1+u_0^n} \end{bmatrix}. \tag{68}$$

The WPT model has the following Jacobian matrix at this steady state:

$$J = \begin{bmatrix} \dfrac{-\left(((2-n)\gamma+3b)\left(\frac{\sigma}{\xi}\right)^{2n}+(b+\gamma)\left(\frac{\sigma}{\xi}\right)^{3n}+((1-n)\gamma+3b)\left(\frac{\sigma}{\xi}\right)^{n}+b\right)(\xi+1)}{\left(\left(\frac{\sigma}{\xi}\right)^{n}+1\right)^2\left((b+\gamma)\left(\frac{\sigma}{\xi}\right)^{n}+b\right)} & b+\dfrac{\gamma\left(\frac{\sigma}{\xi}\right)^{n}}{\left(\frac{\sigma}{\xi}\right)^{n}+1} \\ \dfrac{((2-(\xi+1)n)\gamma+3b)\left(\frac{\sigma}{\xi}\right)^{2n}+(b+\gamma)\left(\frac{\sigma}{\xi}\right)^{3n}+((1-(\xi+1)n)\gamma+3b)\left(\frac{\sigma}{\xi}\right)^{n}+b}{\left(\left(\frac{\sigma}{\xi}\right)^{n}+1\right)^2\left((b+\gamma)\left(\frac{\sigma}{\xi}\right)^{n}+b\right)} & -b-\dfrac{\gamma\left(\frac{\sigma}{\xi}\right)^{n}}{\left(\frac{\sigma}{\xi}\right)^{n}+1} \end{bmatrix} \tag{69}$$

3.4 Hopf bifurcations

Hopf bifurcations occur when complex eigenvalues with real parts greater than zero appear. In this case the real part of the eigenvalues is given by:

$$\mathrm{Re}(\lambda_k) = -\frac{1}{2}k^2(1+D_v)+\frac{1}{2}(f_u+g_v). \tag{70}$$

The value of this real part will keep increasing as k^2 decreases. Since negative values for k^2 don't make sense, the real part will be maximal for $k^2 = 0$ as long as the complex branch exists for this value. Therefore, the maximum real part of λ_k is given by:

$$\max(\mathrm{Re}(\lambda_k)) = \frac{1}{2}(f_u + g_v). \tag{71}$$

This expression can be used to make one parameter Hopf bifurcation plots, with $\max(\mathrm{Re}(\lambda_k))$ plotted against a parameter.

3.5 Two parameter bifurcation analysis

Two parameter bifurcation analysis was performed by repeating a one parameter bifurcation many times for different values of the second parameter and numerically determining at which value(s) of the first parameter $\max(\mathrm{Re}(\lambda_k)) = 0$. To have sufficient sampling points for both parameters, this process was repeated with the two parameters inverted. This process is the same for both Turing and Hopf regimes.

4 Local perturbation analysis

LPA is a relatively new technique for investigating the stability of the homogeneous steady state with respect to an arbitrarily large local perturbation. We will briefly explain it here. A more detailed overview (Edelstein-Keshet et al., 2013), a practical guide (Holmes et al., 2015), and a rigorous mathematical analysis (Holmes, 2014) are available elsewhere. LPA relies on an asymptotic approximation where all rapidly diffusing components are assumed to be infinitely fast ($D \to \infty$) and all slowly diffusing components are assumed to be immobile ($D \to 0$). This way, the evolution of a local pulse in a slowly diffusing activator so narrow that it does not affect global concentrations can be modelled separately from the global level of that activator and any fast components. This reduces the system of PDEs to a system of ODEs, with equations for the local concentration of slow components, the global concentration of slow components and the global concentration of fast components. This system of ODEs can then be analysed using existing bifurcation software to determine the stability of homogeneous (local concentration same as global) and heterogeneous (different local and global concentrations) steady states. Therefore, this method provides similar information to classical LSA (stability of homogeneous states), in addition to information on where a stable homogeneous state could be disrupted by a sufficiently large perturbation.

4.1 LPA of the WP model

The LPA system has ODEs for the local (u_L) and global concentration (u_G) of active GTPase and the global concentration of inactive GTPase (v_G). Since the local perturbation is assumed narrow enough that it does not affect background levels, the global average GTPase concentration $T = u_G + v_G$ is conserved. Using this conservation, we eliminated v_G, leaving the following system:

$$\begin{aligned} \frac{du_L}{dt} &= b(T - u_G) + \gamma \frac{u_L^n}{1 + u_L^n}(T - u_G) - u_L \\ \frac{du_G}{dt} &= b(T - u_G) + \gamma \frac{u_G^n}{1 + u_G^n}(T - u_G) - u_G. \end{aligned} \tag{72}$$

Using continuation software from matlab package matcont, we performed a bifurcation analysis on this system with T as bifurcation parameter (Figure 1). The results revealed three parameter regimes (disregarding Hopf regimes): (1) a regime outside the outermost fold bifurcations of the heterogeneous branch where only a homogeneous state exists, (2) a regime where stable homogeneous and heterogeneous states coexist and a sufficiently large perturbation could switch the system from the homogeneous to the heterogeneous state, and (3) a regime within the two transcritical bifurcations where the homogeneous state is linearly unstable (a Turing regime). To generate the two-parameter bifurcation plots, we performed further continuations of the transcritical bifurcations (black crosses) and outermost fold bifurcations (black dots).

Figure 1 also shows a pair of Hopf bifurcations. Stability does not change in these points and they do not seem to have any effect on the outcome of the simulations of the full PDE system, so we did not consider them any further. For some of the other models (WPT and WP-GAP), Hopf bifurcations were found that changed the stability of the heterogeneous curve from stable to unstable in the region with an unstable homogeneous state. These Hopf bifurcations correspond to those found with LSA and we performed continuations on them to determine the Hopf regimes in the two parameter bifurcation plots.

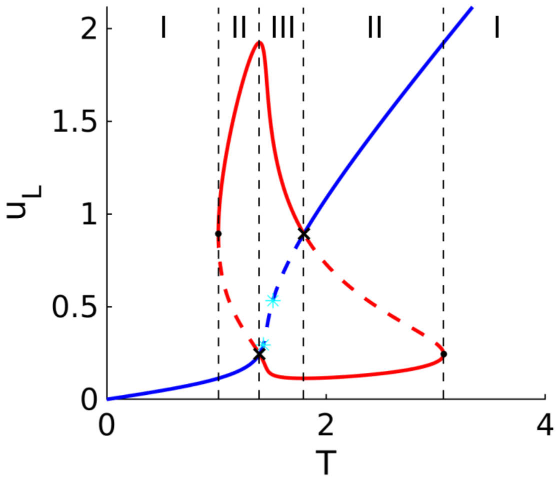

Figure 1: Single parameter bifurcation analysis of the WP LPA system with T as bifurcation parameter and other parameters at default values. The blue curve represents the homogeneous (global) steady state and the red curve the heterogeneous (local) steady state. In regime I, only a homogeneous state exists. In regime II, both a stable homogeneous and heterogeneous steady state exist, so that a sufficiently large perturbation can change the system from a homogeneous to a heterogeneous state. In regime III (Turing regime), the homogeneous state is unstable, so that any small perturbation will result in pattern formation. Solid lines: stable states. Dashed lines: unstable states. Dots: fold bifurcations delimiting the borders of the regime with heterogeneous states. Crosses: transcritical bifurcations delimiting the unstable part of the homogeneous branch (Turing regime). Stars: Hopf bifurcations.

4.2 LPA of the MI model

The LPA method was performed on the MI model as previously described (Holmes and Edelstein-Keshet, 2016). The full LPA ODE system has local and global concentrations for active GTPase 1 and 2 ($u_{1,L}$, $u_{1,G}$, $u_{2,L}$, and $u_{2,G}$) and global concentrations for inactive GTPase 1 and 2 ($v_{1,G}$, and $v_{2,G}$). The global average concentration of GTPase 1 ($T_1 = u_{1,G} + v_{1,G}$) and 2 ($T_2 = u_{2,G} + v_{2,G}$) are conserved, allowing us to eliminate $v_{1,G}$, and $v_{2,G}$. This leads to the

following system of ODEs:

$$\begin{aligned}
\frac{du_{1,L}}{dt} &= b_1(T_1 - u_{1,G}) + \gamma_1 \frac{1}{1+u_{2,L}^n}(T_1 - u_{1,G}) - u_{1,L} \\
\frac{du_{1,G}}{dt} &= b_1(T_1 - u_{1,G}) + \gamma_1 \frac{1}{1+u_{2,G}^n}(T_1 - u_{1,G}) - u_{1,G} \\
\frac{du_{2,L}}{dt} &= b_2(T_2 - u_{2,G}) + \gamma_2 \frac{1}{1+u_{1,L}^n}(T_2 - u_{2,G}) - u_{2,L} \\
\frac{du_{2,G}}{dt} &= b_2(T_2 - u_{2,G}) + \gamma_2 \frac{1}{1+u_{1,G}^n}(T_2 - u_{2,G}) - u_{2,G}.
\end{aligned} \tag{73}$$

A one parameter bifurcation analysis with T_1 as bifurcation parameter and $T_2 = 5$, has a similar result as for the WP model with the same three regimes as for the WP model (Figure 2). We made two parameter bifurcation plots as before by continuation of the bifurcation points delimiting these regimes.

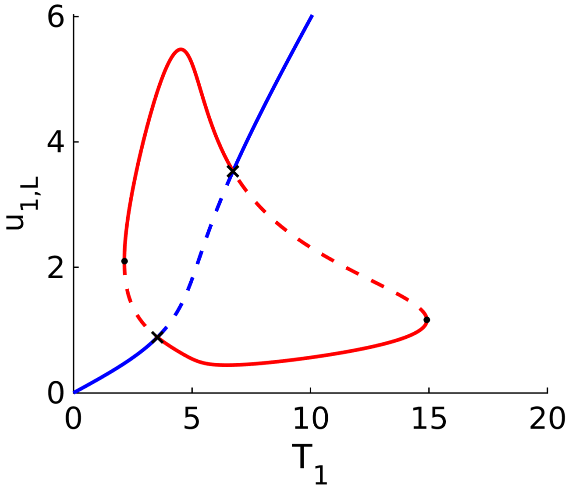

Figure 2: Single parameter bifurcation analysis of the MI LPA system with T_1 as bifurcation parameter, $T_2 = 5$, and other parameters at default values. The blue curve represents the homogeneous (global) steady state and the red curve the heterogeneous (local) steady state. Solid lines: stable states. Dashed lines: unstable states. Dots: fold bifurcations delimiting the borders of the regime with heterogeneous states. Crosses: transcritical bifurcations delimiting the unstable part of the homogeneous branch (Turing regime).

4.3 LPA of the WPT model

The full LPA ODE system of the WPT model has equations for local and global concentrations of active GTPase ($u_{1,L}$ and $u_{1,G}$ respectively) and for the global concentration of inactive GTPase ($v_{1,G}$):

$$\begin{aligned}
\frac{du_L}{dt} &= bv_G + \gamma \frac{u_L^n}{1+u_L^n} v_G - u_L - \xi u_L \\
\frac{du_G}{dt} &= bv_G + \gamma \frac{u_G^n}{1+u_G^n} v_G - u_G - \xi u_G \\
\frac{dv_G}{dt} &= -bv_G - \gamma \frac{u_G^n}{1+u_G^n} v_G + u_G + \sigma.
\end{aligned} \tag{74}$$

Since this model is not mass conserved, none of these equations can be eliminated. A one parameter bifurcation analysis with σ as bifurcation parameter again reveals the same three

regimes as for the WP model (Figure 3). We made two parameter bifurcation plots in the same way as before.

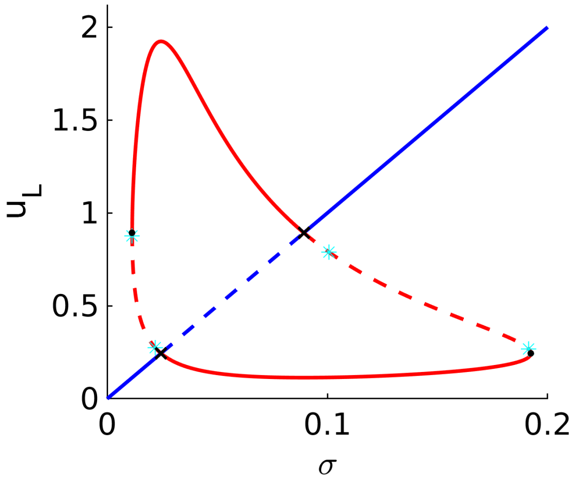

Figure 3: Single parameter bifurcation analysis of the WPT LPA system with σ as bifurcation parameter and other parameters at default values. The blue curve represents the homogeneous (global) steady state and the red curve the heterogeneous (local) steady state. Solid lines: stable states. Dashed lines: unstable states. Dots: fold bifurcations delimiting the borders of the regime with heterogeneous states. Crosses: transcritical bifurcations delimiting the unstable part of the homogeneous branch (Turing regime). Stars: Hopf bifurcations.

4.4 LPA of the MIT model

The full LPA ODE system of the MIT has equations for local and global concentrations of active GTPase 1 and 2 ($u_{1,L}$, $u_{1,G}$, $u_{2,L}$, and $u_{2,G}$) and global concentrations of inactive GTPase 1 and 2 ($v_{1,G}$, and $v_{2,G}$):

$$\begin{aligned}
\frac{du_{1,L}}{dt} &= b_1 v_{1,G} + \gamma_1 \frac{1}{1+u_{2,L}^n} v_{1,G} - u_{1,L} - \xi_1 u_{1,L} \\
\frac{du_{1,G}}{dt} &= b_1 v_{1,G} + \gamma_1 \frac{1}{1+u_{2,G}^n} v_{1,G} - u_{1,G} - \xi_1 u_{1,G} \\
\frac{dv_{1,G}}{dt} &= -b_1 v_{1,G} - \gamma_1 \frac{1}{1+u_{2,G}^n} v_{1,G} + u_{1,G} + \sigma_1 \\
\frac{du_{2,L}}{dt} &= b_2 v_{2,G} + \gamma_2 \frac{1}{1+u_{1,L}^n} v_{2,G} - u_{2,L} - \xi_2 u_{2,L} \\
\frac{du_{2,G}}{dt} &= b_2 v_{2,G} + \gamma_2 \frac{1}{1+u_{1,G}^n} v_{2,G} - u_{2,G} - \xi_2 u_{2,G} \\
\frac{dv_{2,G}}{dt} &= -b_2 v_{2,G} - \gamma_2 \frac{1}{1+u_{1,G}^n} v_{2,G} + u_{2,G} + \sigma_2.
\end{aligned} \tag{75}$$

Since this model is not mass conserved, none of these equations can be eliminated. A one parameter bifurcation analysis with σ_1 as bifurcation parameter and $\sigma_2 = 0.2$ again reveals the same three regimes as for the WP model (Figure 4). We made two parameter bifurcation plots in the same way as before.

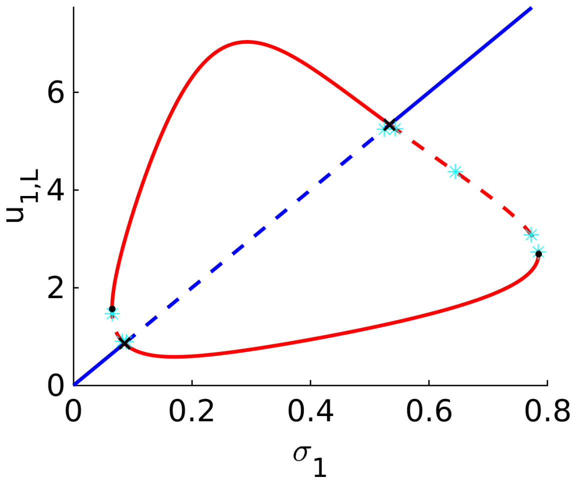

Figure 4: Single parameter bifurcation analysis of the MIT LPA system with σ_1 as bifurcation parameter, $\sigma_2 = 0.2$, and other parameters at default values. The blue curve represents the homogeneous (global) steady state and the red curve the heterogeneous (local) steady state. Solid lines: stable states. Dashed lines: unstable states. Dots: fold bifurcations delimiting the borders of the regime with heterogeneous states. Crosses: transcritical bifurcations delimiting the unstable part of the homogeneous branch (Turing regime). Stars: Hopf bifurcations.

4.5 LPA of the WPGAP model

We performed LPA both with fast diffusing and slowly diffusing active GAP. For fast diffusing active GAP, the LPA system has ODEs for the local and global concentration of active GTPase (u_L and u_G respectively) and the global concentration of inactive GTPase, active GAP, and inactive GAP (v_G, G_G, and g_G respectively). The global average GTPase concentration $T = u_G + v_G$ and GAP concentration $T_g = G_G + g_G$ are conserved. Using these conservations, we eliminated v_G and g_G, leaving the following system:

$$\begin{aligned}
\frac{du_L}{dt} &= b(T - u_G) + \gamma \frac{u_L^n}{1 + u_L^n}(T - u_G) - u_L - G_G \cdot u_L \\
\frac{du_G}{dt} &= b(T - u_G) + \gamma \frac{u_G^n}{1 + u_G^n}(T - u_G) - u_G - G_G \cdot u_G \\
\frac{dG_G}{dt} &= cu_G(T_g - G_G) - dG_G.
\end{aligned} \tag{76}$$

For slowly diffusing active GAP, an additional local concentration of active GAP (G_L) has to be added to the LPA system:

$$\begin{aligned}
\frac{du_L}{dt} &= b(T - u_G) + \gamma \frac{u_L^n}{1 + u_L^n}(T - u_G) - u_L - G_L \cdot u_L \\
\frac{du_G}{dt} &= b(T - u_G) + \gamma \frac{u_G^n}{1 + u_G^n}(T - u_G) - u_G - G_G \cdot u_G \\
\frac{dG_L}{dt} &= cu_L(T_g - G_G) - dG_L \\
\frac{dG_G}{dt} &= cu_G(T_g - G_G) - dG_G.
\end{aligned} \tag{77}$$

A one parameter bifurcation analysis with T as bifurcation parameter again reveals the same three regimes for the system with fast active GAP as for the WP model (Figure 5). For the system with slow active GAP there is no heterogeneous state at default T_g. For lower values

of T_g, the three regimes can still be found for this system (Figure 5). We made two parameter bifurcation plots in the same way as before.

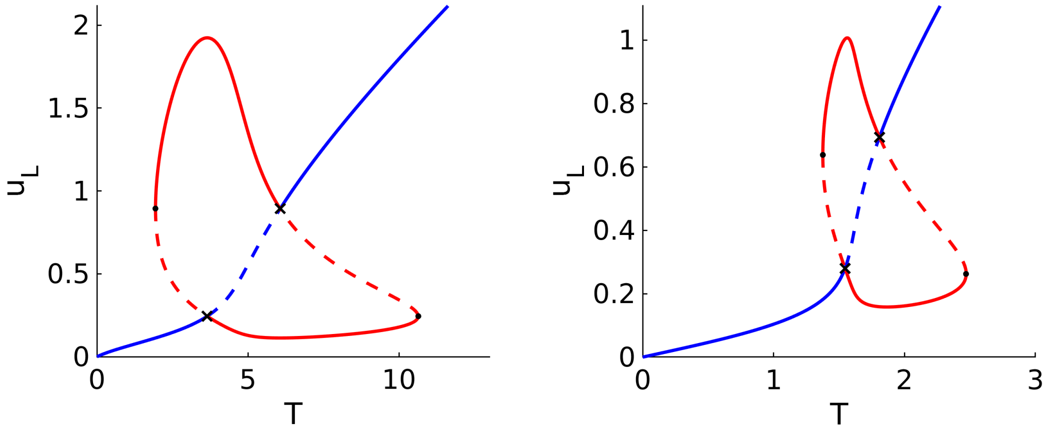

Figure 5: Single parameter bifurcation analysis of the WPGAP LPA system with T as bifurcation parameter and other parameters at default values. Left: LPA system for fast active GAP. Right: LPA system for slow active GAP (with $T_g = 0.5$). The blue curve represents the homogeneous (global) steady state and the red curve the heterogeneous (local) steady state. Solid lines: stable states. Dashed lines: unstable states. Dots: fold bifurcations delimiting the borders of the regime with heterogeneous states. Crosses: transcritical bifurcations delimiting the unstable part of the homogeneous branch (Turing regime).

4.6 LPA of the MIGAP1 model

Based on our results for the WPGAP model, we performed LPA on the MI models with GAPs only for fast diffusing GAPs. The full LPA ODE system has local and global concentrations for active GTPase 1 and 2 ($u_{1,L}$, $u_{1,G}$, $u_{2,L}$, and $u_{2,G}$) and global concentrations for inactive GTPase 1 and 2 ($v_{1,G}$, and $v_{2,G}$) and active and inactive GAP ($G_{1,G}$ and $g_{1,G}$). The global average concentration of GTPase 1 ($T_1 = u_{1,G} + v_{1,G}$) and 2 ($T_2 = u_{2,G} + v_{2,G}$) and GAP ($T_{g,1} = G_{1,G} + g_{1,G}$) are conserved, allowing us to eliminate $v_{1,G}$, $v_{2,G}$, and $g_{1,G}$. This leads to the following system of ODEs:

$$
\begin{aligned}
\frac{du_{1,L}}{dt} &= b_1(T_1 - u_{1,G}) + \gamma_1 \frac{1}{1+u_{2,L}^n}(T_1 - u_{1,G}) - u_{1,L} - G_{1,G} \cdot u_{1,L} \\
\frac{du_{1,G}}{dt} &= b_1(T_1 - u_{1,G}) + \gamma_1 \frac{1}{1+u_{2,G}^n}(T_1 - u_{1,G}) - u_{1,G} - G_{1,G} \cdot u_{1,G} \\
\frac{du_{2,L}}{dt} &= b_2(T_2 - u_{2,G}) + \gamma_2 \frac{1}{1+u_{1,L}^n}(T_2 - u_{2,G}) - u_{2,L} \\
\frac{du_{2,G}}{dt} &= b_2(T_2 - u_{2,G}) + \gamma_2 \frac{1}{1+u_{1,G}^n}(T_2 - u_{2,G}) - u_{2,G} \\
\frac{dG_{1,G}}{dt} &= c_1 u_{1,G}(T_{g,1} - g_{1,G}) - d_1 G_{1,G}.
\end{aligned}
\tag{78}
$$

A one parameter bifurcation analysis with T_1 as bifurcation parameter and $T_2 = 5$ again reveals the same three regimes as for the WP model (Figure 6). We made two parameter bifurcation plots in the same way as before.

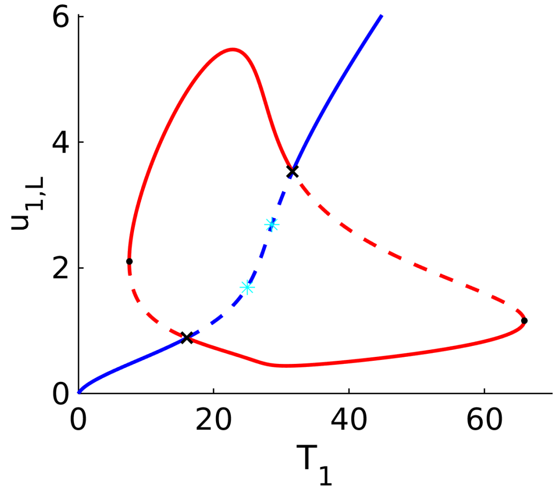

Figure 6: Single parameter bifurcation analysis of the MIGAP1 LPA system with T_1 as bifurcation parameter, $T_2 = 5$, and other parameters at default values. The blue curve represents the homogeneous (global) steady state and the red curve the heterogeneous (local) steady state. Solid lines: stable states. Dashed lines: unstable states. Dots: fold bifurcations delimiting the borders of the regime with heterogeneous states. Crosses: transcritical bifurcations delimiting the unstable part of the homogeneous branch (Turing regime). Stars: Hopf bifurcations.

4.7 LPA of the MIGAP2 model

The full LPA ODE system has local and global concentrations for active GTPase 1 and 2 ($u_{1,L}$, $u_{1,G}$, $u_{2,L}$, and $u_{2,G}$) and global concentrations for inactive GTPase 1 and 2 ($v_{1,G}$, and $v_{2,G}$) and acitve and inactive GAP 1 and 2 ($G_{1,G}$, $g_{1,G}$, $G_{2,G}$, and $g_{2,G}$). The global average concentration of GTPase 1 ($T_1 = u_{1,G} + v_{1,G}$) and 2 ($T_2 = u_{2,G} + v_{2,G}$) and GAP 1 ($T_{g,1} = G_{1,G} + g_{1,G}$) and 2 ($T_{g,2} = G_{2,G} + g_{2,G}$) are conserved, allowing us to eliminate $v_{1,G}$, $v_{2,G}$, $g_{1,G}$, and $g_{2,G}$. This leads to the following system of ODEs:

$$
\begin{aligned}
\frac{du_{1,L}}{dt} &= b_1(T_1 - u_{1,G}) + \gamma_1 \frac{1}{1 + u_{2,L}^n}(T_1 - u_{1,G}) - u_{1,L} - G_{1,G} \cdot u_{1,L} \\
\frac{du_{1,G}}{dt} &= b_1(T_1 - u_{1,G}) + \gamma_1 \frac{1}{1 + u_{2,G}^n}(T_1 - u_{1,G}) - u_{1,G} - G_{1,G} \cdot u_{1,G} \\
\frac{du_{2,L}}{dt} &= b_2(T_2 - u_{2,G}) + \gamma_2 \frac{1}{1 + u_{1,L}^n}(T_2 - u_{2,G}) - u_{2,L} - G_{2,G} \cdot u_{2,L} \\
\frac{du_{2,G}}{dt} &= b_2(T_2 - u_{2,G}) + \gamma_2 \frac{1}{1 + u_{1,G}^n}(T_2 - u_{2,G}) - u_{2,G} - G_{2,G} \cdot u_{2,G} \\
\frac{dG_{1,G}}{dt} &= c_1 u_{1,G}(T_{g,1} - G_{1,G}) - d_1 G_{1,G} \\
\frac{dG_{2,G}}{dt} &= c_2 u_{2,G}(T_{g,2} - G_{2,G}) - d_2 G_{2,G}.
\end{aligned}
\tag{79}
$$

A one parameter bifurcation analysis with T_1 as bifurcation parameter and $T_2 = 20$ again reveals the same three regimes as for the WP model (Figure 7). We made two parameter bifurcation plots in the same way as before.

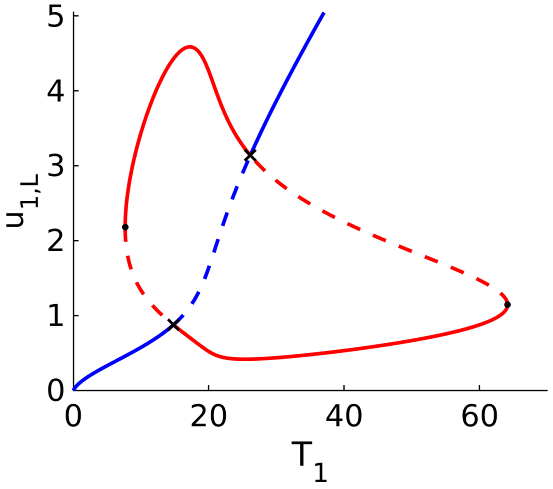

Figure 7: Single parameter bifurcation analysis of the MIGAP2 LPA system with T_1 as bifurcation parameter, $T_2 = 20$, and other parameters at default values. The blue curve represents the homogeneous (global) steady state and the red curve the heterogeneous (local) steady state. Solid lines: stable states. Dashed lines: unstable states. Dots: fold bifurcations delimiting the borders of the regime with heterogeneous states. Crosses: transcritical bifurcations delimiting the unstable part of the homogeneous branch (Turing regime).

5 WPT model with degradation of inactive GTPase

If the degradation term from the WPT model is changed from degradation of active GTPase to degradation of inactive GTPase, the full dimension-carrying system is given by:

$$\begin{aligned} \frac{\partial \tilde{u}}{\partial \tilde{t}} &= \tilde{b}\tilde{v} + \tilde{\gamma}\frac{\tilde{u}^n}{K^n + \tilde{u}^n}\tilde{v} - \delta\tilde{u} + D_u\nabla^2\tilde{u} \\ \frac{\partial \tilde{v}}{\partial \tilde{t}} &= -\tilde{b}\tilde{v} - \tilde{\gamma}\frac{\tilde{u}^n}{K^n + \tilde{u}^n}\tilde{v} + \delta\tilde{u} + \tilde{\sigma} - \tilde{\xi}\tilde{v} + \tilde{D}_v\nabla^2\tilde{v}. \end{aligned} \tag{80}$$

After non-dimensionalisation, the following system is obtained:

$$\begin{aligned} \frac{\partial u}{\partial t} &= bv + \gamma\frac{u^n}{1 + u^n}v - u + \nabla^2 u \\ \frac{\partial v}{\partial t} &= -bv - \gamma\frac{u^n}{1 + u^n}v + u + \sigma - \xi v + D_v\nabla^2 v, \end{aligned} \tag{81}$$

with dimensionless variables:

$$u = \frac{\tilde{u}}{K}, \quad v = \frac{\tilde{v}}{K}, \quad t = \delta\tilde{t}, \quad x = \frac{\tilde{x}\sqrt{\delta}}{\sqrt{D_u}}, \quad y = \frac{\tilde{y}\sqrt{\delta}}{\sqrt{D_u}}, \tag{82}$$

and scaled parameters:

$$b = \frac{\tilde{b}}{\delta}, \quad \gamma = \frac{\tilde{\gamma}}{\delta}, \quad \xi = \frac{\tilde{\xi}}{\delta}, \quad \sigma = \frac{\tilde{\sigma}}{\delta K}, \quad D_v = \frac{\tilde{D}_v}{D_u}. \tag{83}$$

The corresponding LPA system is:

$$\begin{aligned} \frac{du_L}{dt} &= bv_G + \gamma\frac{u_L^n}{1 + u_L^n}v_G - u_L \\ \frac{du_G}{dt} &= bv_G + \gamma\frac{u_G^n}{1 + u_G^n}v_G - u_G \\ \frac{dv_G}{dt} &= -bv_G - \gamma\frac{u_G^n}{1 + u_G^n}v_G + u_G + \sigma - \xi v_G. \end{aligned} \tag{84}$$

A one parameter bifurcation analysis with σ as bifurcation parameter reveals a bistable regime with an unstable homogeneous state surrounded by two stable homogeneous steady states (Fig 8). The heterogeneous steady states follow the homogeneous ones so closely that the order of the curves could not be resolved numerically. Therefore, we further studied the behaviour of this system with a numerical simulation starting somewhere in the middle of the unstable part of the homogeneous branch. In this simulation, many clusters of active GTPase initially appear and start growing. However, they keep growing until they fill the domain and a new homogeneous state is reached (Fig 9), indicating that the unusual heterogeneous branch in the LPA plot does not represent a reachable heterogeneous state.

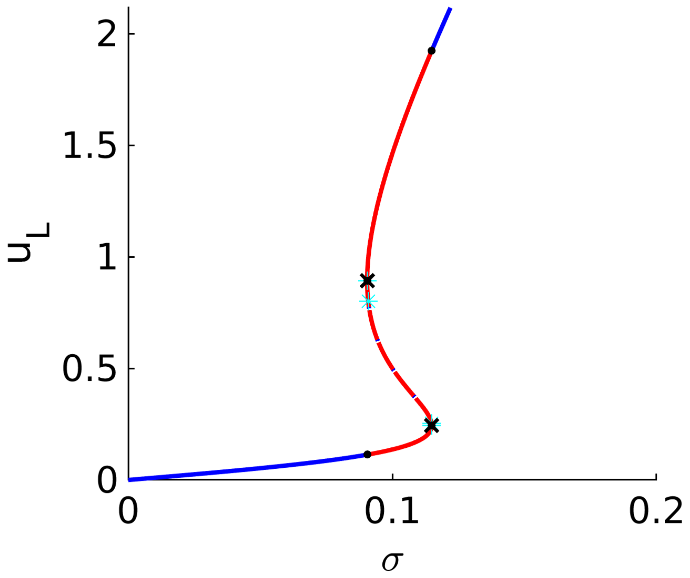

Figure 8: Single parameter bifurcation analysis of the WPT LPA system with degradation of inactive GTPase with σ as bifurcation parameter and other parameters at default values. The blue curve represents the homogeneous (global) steady state and the red curve the heterogeneous (local) steady state. Inset shows details around $u_L = 0.5$. Solid lines: stable states. Dashed lines: unstable states. Dots: fold bifurcations delimiting the borders of the regime with heterogeneous states. Crosses: transcritical bifurcations delimiting the unstable part of the homogeneous branch (Turing regime). Stars: Hopf bifurcations.

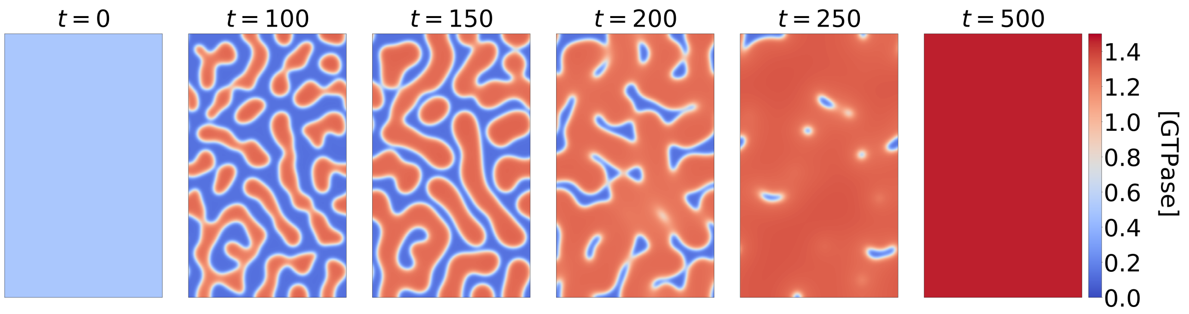

Figure 9: Transient active GTPase concentrations ([GTPase]) from simulations of the WPT model with degradation of the inactive form of GTPase. Simulation conditions were as described in the main text for the WPT model, with $\sigma = 0.1$.

Attempts with different parameters in the LPA system did not yield any bifurcation plots with clearly reachable heterogeneous states, indicating that it is at the vary least easier to obtain spontaneous pattern formation when degradation of the active form occurs. This suggests some

removal of active GTPase (e.g. by membrane recycling) is important for pattern formation mechanisms with broken mass conservation.

6 Derivation of ODE models

6.1 Basic ODE model

The homogeneous WP system is bistable, with points within the cluster being in the high steady state and points outside the cluster in the low steady sate (Mori et al., 2008). Similarly, for the MI model, the area outside a cluster of GTPase 1 is dominated by GTPase 2 which promotes inactivation of GTPase 1. This property results in a relatively flat concentration profile inside clusters. We approximate this by a single, constant (high) concentration inside clusters and a single, constant (low) concentration outside clusters (Fig 10).

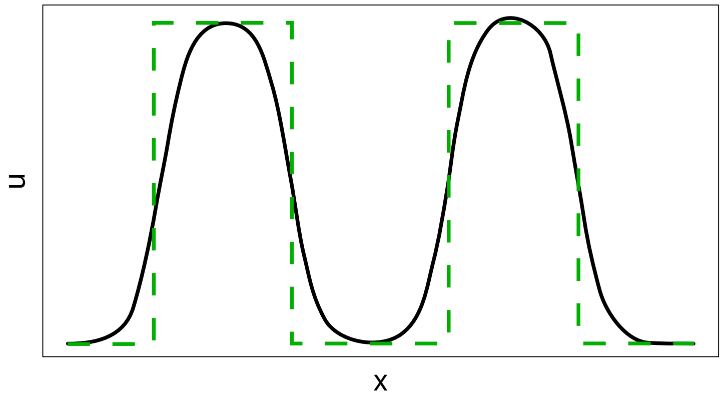

Figure 10: Cartoon of cluster representation used in the ODE model. The solid black line represents a profile of the active GTPase concentration (u) as obtained with the PDE model along a 1D cross-section in direction x. The dashed green line indicates a discrete cluster approximation based on this profile.

Under this approximation, cluster area is proportional to the amount of (active) GTPase $h_{c,n}$ in cluster n and cluster circumference is proportional to $h_{c,n}^{\eta}$ with $0 < \eta < 1$, depending on cluster shape (and system dimensionality). For circular clusters, $\eta = \frac{1}{2}$ because the area is directly proportional to the square root of the circumference. In addition, we assume diffusion of the inactive form is sufficiently fast that all clusters effectively draw from a single pool of inactive GTPase. Due to the constant area of the membrane, the constant inactive GTPase concentration v is directly proportional to the total amount of inactive GTPase h_p. Since the concentration of active GTPase in the clusters is assumed constant ($u = u_c$), the interaction function f of the WP model can be simplified:

$$f(u, v) = bv + \gamma \frac{u^n}{1 + u^n} v - u = \left(b + \gamma \frac{u_c^n}{1 + u_c^n} \right) v - u_c = c_1 h_p - c_2, \qquad (85)$$

where c_1 and c_2 are constants. Similarly, interaction function f of the MI model can be simplified to obtain the same expression if we assume levels of active GTPase outside clusters to also be approximately constant:

$$f(u_i, v_i, u_j) = b_i v_i + \gamma_i \frac{1}{1 + u_j^n} v_i - u_i = \left(b_i + \gamma_i \frac{1}{1 + u_{j,c}^n} \right) v_i - u_{i,c} = c_1 h_{p,i} - c_2. \qquad (86)$$

Since these functions describe the change of concentrations and we are interested in cluster size, we have to integrate this expression across the area $A_{c,n}$ of the cluster, which is proportional to $h_{c,n}$. Because inactive GTPase concentration v is homogeneous, this integral is easily solved:

$$\iint_{A_{c,n}} (c_1 h_p - c_2) dA_{c,n} = (c_1 h_p - c_2) \iint_{A_{c,n}} dA_{c,n} = c_1 A_{c,n} h_p - c_2 A_{c,n} = \alpha h_{c,n} h_p - \delta h_{c,n}, \tag{87}$$

where α and δ are constants. These terms describe changes in cluster sizes due to the interaction functions.

In addition to these terms, we consider loss of active GTPase through diffusion across the boundary of the cluster, where we assume it is immediately converted into the inactive form because of the low steady state of the active form outside the cluster. This results in an extra inactivation term proportional to the circumference of the cluster $h_{c,n}^{\eta}$. Combining this term (using proportionality constant β) with the terms derived from the interaction function results in the basic ODE model:

$$\begin{aligned} \frac{dh_{c,n}(t)}{dt} &= \alpha h_{c,n}(t) h_p(t) - \beta h_{c,n}(t)^{\eta} - \delta h_{c,n}(t) \\ \frac{dh_p(t)}{dt} &= -\alpha h_p(t) \sum_{j=1}^{N} h_{c,j}(t) + \beta \sum_{j=1}^{N} h_{c,j}(t)^{\eta} + \delta \sum_{j=1}^{N} h_{c,j}(t). \end{aligned} \tag{88}$$

6.2 Compartmentalised ODE model

We account for the fact that a cluster can only incorporate inactive GTPase from its vicinity by giving each cluster its own local compartment containing an amount of inactive GTPase $h_{p,n}$. We can greatly simplify computations by assuming that all compartments have equal size and are connected to all other compartments with equally large interfaces. This is a reasonable approximation for the question we are most interested in: do small differences in cluster size increase (polarisation) or decrease (stable coexistence)? Under this approximation, diffusive fluxes of inactive GTPase become constant rates of exchange between compartments.

Upon adding a production term to the inactive form and degradation terms to both the active and inactive forms, the system of PDEs reads:

$$\begin{aligned} \frac{\partial u}{\partial t} &= f(u, v) - \xi u \\ \frac{\partial v}{\partial t} &= -f(u, v) + \sigma - \xi_p v. \end{aligned} \tag{89}$$

To obtain the terms of the ODE approximation, the extra terms can be treated as done before for function f. Since compartments have a constant size, concentration v in each compartment is proportional to $h_{p,n}$. In clusters, concentration u is still constant. Dropping function f for the moment, we get:

$$\begin{aligned} \frac{\partial u}{\partial t} &= -\xi u_c = -c_3 \\ \frac{\partial v}{\partial t} &= \sigma - c_4 h_{p,n}, \end{aligned} \tag{90}$$

where c_3 and c_4 are constants. To obtain expressions for $h_{c,n}$ and $h_{p,n}$, we integrate across the cluster area $A_{c,n}$ (for degradation of active form), or the compartment area $A_{p,n}$ (for production

and degradation of inactive form):

$$
\begin{aligned}
\frac{dh_{c,n}}{dt} &= \iint_{A_{c,n}} -c_3 dA_{c,n} = -c_3 A_{c,n} = -\tilde{\xi} h_{c,n} \\
\frac{dh_{p,n}}{dt} &= \iint_{A_{p,n}} (\sigma - c_4 h_{p,n}) dA_{p,n} = \sigma A_{p,n} - c_4 A_{p,n} h_{p,n} = \tilde{\sigma} - \tilde{\xi}_p h_{p,n},
\end{aligned}
\tag{91}
$$

where $\tilde{\xi}$, $\tilde{\sigma}$, and $\tilde{\xi}_p$ are constants. Adding these terms and a constant exchange rate ϕ to the expression we already found in the previous section (and dropping the tildes) we obtain the compartmentalised ODE system:

$$
\begin{aligned}
\frac{dh_{c,n}(t)}{dt} &= \alpha h_{c,n}(t) h_{p,n}(t) - \beta h_{c,n}(t)^{\eta} - \delta h_{c,n}(t) - \xi h_{c,n}(t) \\
\frac{dh_{p,n}(t)}{dt} &= \sigma - \alpha h_{p,n}(t) h_{c,n}(t) + \beta h_{c,n}(t)^{\eta} + \delta h_{c,n}(t) - \xi_p h_{p,n}(t) + \phi \sum_{j=1}^{N} (h_{p,j} - h_{p,n}).
\end{aligned}
\tag{92}
$$

6.3 ODE model with GAP feedback

If we write $G_{c,n}$ for the total amount of active GAP in cluster n, then the concentration of active GAP in that cluster is proportional to $G_{c,n}/h_{c,n}$. Since $h_{c,n}$ is proportional to both the area of and the total amount of GTPase in cluster n, the GTPase concentration in that cluster is proportional to $h_{c,n}/h_{c,n}$ (constant). Since we modelled GAP-based GTPase inactivation proportional to the product of GAP and GTPase concentrations, the rate at which GAPs reduce the total amount of GTPase in cluster n is proportional to $G_{c,n}/h_{c,n} \cdot h_{c,n}/h_{c,n} \cdot h_{c,n} = G_{c,n}$. Loss of GAPs from the cluster by diffusion is proportional to the GAP concentration and the circumference of the cluster and therefore to $G_{c,n}/h_{c,n} \cdot h_{c,n}^{\eta} = h_{c,n}^{\eta-1} G_{c,n}$. GAPs lost by diffusion are assumed to be inactivated outside the cluster. In addition, GAPs are inactivated inside the cluster at a constant rate, so proportional to the GAP concentration $G_{c,n}/h_{c,n}$. This makes the change in the amount of active GAP due to constant inactivation proportional to $G_{c,n}/h_{c,n} \cdot h_{c,n} = G_{c,n}$. GTPase-dependent GAP activation was modelled proportional to the active GTPase and inactive GAP concentrations in the PDE model. Therefore, the rate at which GTPase inactivates GAP in a cluster is proportional to the (constant) GTPase concentration and the non-cluster inactive GAP concentration G_p (in a constant area). This results in a total GAP activation proportional to $h_{c,n}/h_{c,n} \cdot G_p \cdot h_{c,n} = h_{c,n} G_p$. Combining these relations with the relevant rate constants results in the system of the ODE model with GAP feedback:

$$
\begin{aligned}
\frac{dh_{c,n}(t)}{dt} &= \alpha h_{c,n}(t) h_p(t) - \beta h_{c,n}(t)^{\eta} - \delta h_{c,n}(t) - \varepsilon G_{c,n}(t) \\
\frac{dh_p(t)}{dt} &= -\alpha h_p(t) \sum_{j=1}^{N} h_{c,j}(t) + \beta \sum_{j=1}^{N} h_{c,j}(t)^{\eta} + \delta \sum_{j=1}^{N} h_{c,j}(t) + \varepsilon \sum_{j=1}^{N} G_{c,j}(t) \\
\frac{dG_{c,n}(t)}{dt} &= \gamma h_{c,n}(t) G_p(t) - \delta_g G_{c,n}(t) - \zeta h_{c,n}(t)^{\eta-1} G_{c,n} \\
\frac{dG_p(t)}{dt} &= -\gamma G_p(t) \sum_{j=1}^{N} h_{c,j}(t) + \delta_g \sum_{j=1}^{N} G_{c,j}(t) + \zeta \sum_{j=1}^{N} h_{c,j}(t)^{\eta-1} G_{c,j}(t).
\end{aligned}
\tag{93}
$$

7 WPT model with degradation of both active and inactive GTPase

Since degradation of inactive GTPase only does not seem to yield any stable patterns, we ask if some degree of degradation of inactive GTPase can be tolerated as long as there is also degradation of active GTPase. Simulations with the compartmentalised ODE model with degradation of both active and inactive forms suggests that patterns of stably coexisting GTPase clusters are indeed possible (Fig 11A). To confirm this, we consider the WPT model is with degradation of both active and inactive GTPase. The full dimension-carrying system is given by:

$$\begin{aligned}\frac{\partial \tilde{u}}{\partial \tilde{t}} &= \tilde{b}\tilde{v} + \tilde{\gamma}\frac{\tilde{u}^n}{K^n + \tilde{u}^n}\tilde{v} - \delta\tilde{u} - \tilde{\xi}\tilde{u} + D_u\nabla^2\tilde{u} \\ \frac{\partial \tilde{v}}{\partial \tilde{t}} &= -\tilde{b}\tilde{v} - \tilde{\gamma}\frac{\tilde{u}^n}{K^n + \tilde{u}^n}\tilde{v} + \delta\tilde{u} + \tilde{\sigma} - \tilde{\xi}_p\tilde{v} + \tilde{D}_v\nabla^2\tilde{v}.\end{aligned} \tag{94}$$

After non-dimensionalisation, the following system is obtained:

$$\begin{aligned}\frac{\partial u}{\partial t} &= bv + \gamma\frac{u^n}{1 + u^n}v - u - \xi u + \nabla^2 u \\ \frac{\partial v}{\partial t} &= -bv - \gamma\frac{u^n}{1 + u^n}v + u + \sigma - \xi_p v + D_v\nabla^2 v,\end{aligned} \tag{95}$$

with dimensionless variables:

$$u = \frac{\tilde{u}}{K}, \quad v = \frac{\tilde{v}}{K}, \quad t = \delta\tilde{t}, \quad x = \frac{\tilde{x}\sqrt{\delta}}{\sqrt{D_u}}, \quad y = \frac{\tilde{y}\sqrt{\delta}}{\sqrt{D_u}}, \tag{96}$$

and scaled parameters:

$$b = \frac{\tilde{b}}{\delta}, \quad \gamma = \frac{\tilde{\gamma}}{\delta}, \quad \xi = \frac{\tilde{\xi}}{\delta}, \quad \xi_p = \frac{\tilde{\xi}_p}{\delta}, \quad \sigma = \frac{\tilde{\sigma}}{\delta K}, \quad D_v = \frac{\tilde{D}_v}{D_u}. \tag{97}$$

The corresponding LPA system is:

$$\begin{aligned}\frac{du_L}{dt} &= bv_G + \gamma\frac{u_L^n}{1 + u_L^n}v_G - u_L - \xi u_L \\ \frac{du_G}{dt} &= bv_G + \gamma\frac{u_G^n}{1 + u_G^n}v_G - u_G - \xi u_G \\ \frac{dv_G}{dt} &= -bv_G - \gamma\frac{u_G^n}{1 + u_G^n}v_G + u_G + \sigma - \xi_p v_G.\end{aligned} \tag{98}$$

A one parameter bifurcation analysis with σ as bifurcation parameter reveals three parameter regimes: a homogeneous regime, a Turing regime, and a regime where patterning may be induced by a sufficiently strong perturbation (Fig 11B). A simulation in the Turing regime reveals that this model can indeed yield a pattern of multiple stably coexisting clusters (Fig 11C).

8 Quasi steady state approximation for GAPs in ODE model

Due to mass conservation, the equation for the amount of (inactive) GAP G_p outside the cluster is redundant. Using the conservation relation $T_g = G_p + \sum_{j=1}^{N} G_{c,j}$, this equation can be

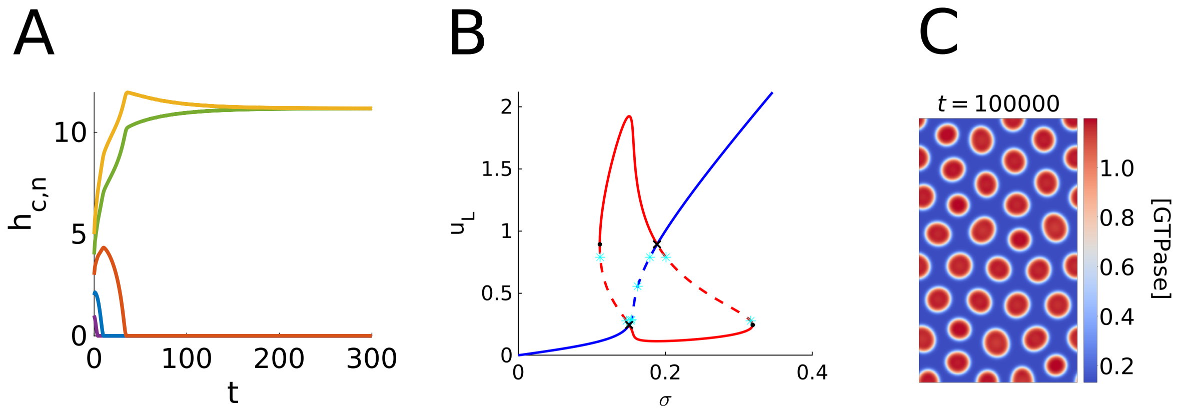

Figure 11: Coexistence can be found even with degradation of both active and inactive forms. A: Simulation of compartmentalised ODE model with production and degradation. Parameters were as in main text, with $\phi = 1$ and $\xi = \xi_p = 0.1$. B: Single parameter bifurcation analysis of the WPT model with an additional degradation of inactive form at the same rate as the active form. Colours and symbols are as in Fig 8. C: Profile of steady active GTPase concentrations ([GTPase]) from simulations of the WPT model with degradation of both the active and inactive form of GTPase. Simulation conditions were as described in the main text for the WPT model, with $\sigma = 0.155$ and a degradation rate of 0.1 for both active and inactive forms.

removed from the ODE system, leaving for GAP dynamics only the equations for $G_{c,n}$:

$$\frac{dG_{c,n}(t)}{dt} = \gamma h_{c,n}(t)\left(T_g - \sum_{j=1}^{N} G_{c,j}(t)\right) - \zeta G_{c,n}(t) h_{c,n}(t)^{\eta-1} - \delta_g G_{c,n}(t). \tag{99}$$

In the case where GAP dynamics is fast compared to GTPase dynamics, we can take a quasi steady state approximation for the amounts of GAP. This results in:

$$G_{c,n} = \frac{\gamma h_{c,n}(t)}{\zeta h_{c,n}(t)^{\eta-1} + \delta_g}\left(T_g - \sum_{j=1}^{N} G_{c,j}\right). \tag{100}$$

To obtain an expression for $\sum_{j=1}^{N} G_{c,j}$, we sum $G_{c,n}$ over all clusters:

$$\begin{aligned}\sum_{j=1}^{N} G_{c,j} = \sum_{n=1}^{N} G_{c,n} &= \sum_{n=1}^{N} \frac{\gamma h_{c,n}(t)}{\zeta h_{c,n}(t)^{\eta-1} + \delta_g}\left(T_g - \sum_{j=1}^{N} G_{c,j}\right) \\ &= \left(T_g - \sum_{j=1}^{N} G_{c,j}\right)\sum_{n=1}^{N} \frac{\gamma h_{c,n}(t)}{\zeta h_{c,n}(t)^{\eta-1} + \delta_g}.\end{aligned} \tag{101}$$

Solving for $\sum_{j=1}^{N} G_{c,j}$ and (trivially) changing summation indices gives:

$$\sum_{j=1}^{N} G_{c,j} = T_g \frac{\sum_{j=1}^{N} \dfrac{h_{c,j}(t)}{h_{c,j}(t)^{\eta-1} + \delta_g/\zeta}}{\zeta/\gamma + \sum_{j=1}^{N} \dfrac{h_{c,j}(t)}{h_{c,j}(t)^{\eta-1} + \delta_g/\zeta}} \tag{102}$$

Substituting this expression into Eq 100 gives:

$$\begin{aligned}
G_{c,n} &= \frac{\gamma h_{c,n}(t)}{\zeta h_{c,n}(t)^{\eta-1}+\delta_g}\left(T_g - T_g\frac{\sum_{j=1}^{N}\dfrac{h_{c,j}(t)}{h_{c,j}(t)^{\eta-1}+\delta_g/\zeta}}{\zeta/\gamma+\sum_{j=1}^{N}\dfrac{h_{c,j}(t)}{h_{c,j}(t)^{\eta-1}+\delta_g/\zeta}}\right) \\
&= T_g\frac{\gamma h_{c,n}(t)}{\zeta h_{c,n}(t)^{\eta-1}+\delta_g}\cdot\frac{\zeta/\gamma}{\zeta/\gamma+\sum_{j=1}^{N}\dfrac{h_{c,j}(t)}{h_{c,j}(t)^{\eta-1}+\delta_g/\zeta}} \\
&= T_g\frac{\dfrac{h_{c,n}(t)}{h_{c,n}(t)^{\eta-1}+\delta_g/\zeta}}{\zeta/\gamma+\sum_{j=1}^{N}\dfrac{h_{c,j}(t)}{h_{c,j}(t)^{\eta-1}+\delta_g/\zeta}}.
\end{aligned} \tag{103}$$

We now have an expression for $G_{c,n}$ that only depends on $h_{c,n}$ and other $h_{c,i}$. With this we can close the equations for $h_{c,n}$ in terms of h_c.

References

Edelstein-Keshet, L., Holmes, W. R., Zajac, M., and Dutot, M. (2013). From simple to detailed models for cell polarization. *Philosophical Transactions of the Royal Society of London B: Biological Sciences*, 368(1629).

Holmes, W. R. (2014). An efficient, nonlinear stability analysis for detecting pattern formation in reaction diffusion systems. *Bulletin of Mathematical Biology*, 76(1):157–183.

Holmes, W. R. and Edelstein-Keshet, L. (2016). Analysis of a minimal Rho-GTPase circuit regulating cell shape. *Physical Biology*, 13(4):046001.

Holmes, W. R., Mata, M. A., and Edelstein-Keshet, L. (2015). Local perturbation analysis: A computational tool for biophysical reaction-diffusion models. *Biophysical Journal*, 108(2):230 – 236.

Miura, T. and Maini, P. K. (2004). Speed of pattern appearance in reaction-diffusion models: Implications in the pattern formation of limb bud mesenchyme cells. *Bulletin of Mathematical Biology*, 66(4):627–649.

Mori, Y., Jilkine, A., and Edelstein-Keshet, L. (2008). Wave-pinning and cell polarity from a bistable reaction-diffusion system. *Biophysical Journal*, 94(9):3684 – 3697.

Murray, J. D. (2001). *Mathematical Biology. II Spatial Models and Biomedical Applications* {*Interdisciplinary Applied Mathematics V. 18*}. Springer-Verlag New York Incorporated.
